# Supplementary material for: Evidence of bifunctionality of carbons and metal atoms in catalyzed acetylene hydrochlorination
Source: Nat Commun. 2023 Sep 9;14:5557. doi: 10.1038/s41467-023-41344-0 (PMC10492806; doi:10.1038/s41467-023-41344-0)
Supplement: Supplementary file 1 — Supplementary Information [file 41467_2023_41344_MOESM1_ESM.pdf]

Supplementary Information

Evidence of bifunctionality of carbons and metal atoms in catalyzed acetylene hydrochlorination

Vera Giulimondi[1], Andrea Ruiz-Ferrando[2,3], Georgios Giannakakis[1], Ivan Surin[1], Mikhail Agrachev[4], Gunnar Jeschke[4], Frank Krumeich[5], Núria López[2], Adam H. Clark[6], and Javier Pérez-Ramírez[1]*

[1] Institute for Chemical and Bioengineering, Department of Chemistry and Applied Biosciences, ETH Zurich, Vladimir-Prelog-Weg 1, 8093 Zurich, Switzerland.

[2] Institute of Chemical Research of Catalonia (ICIQ), Av. Països Catalans 16, Tarragona 43007, Spain.

[3] Department of Physical and Inorganic Chemistry, Universitat Rovira i Virgili, Marcel·lí Domingo s/n, Tarragona 43007, Spain.

[4] Laboratory of Physical Chemistry, Department of Chemistry and Applied Biosciences, ETH Zurich, Vladimir-Prelog-Weg 1, 8093 Zurich, Switzerland.

[5] Laboratory of Inorganic Chemistry, Department of Chemistry and Applied Biosciences, ETH Zurich, Vladimir-Prelog-Weg 1, 8093 Zurich, Switzerland.

[6] Paul Scherrer Institute, 5232 Villigen PSI, Switzerland.

* Corresponding author. E-mail: jpr@chem.ethz.ch

Table of Contents

1. Supplementary Methods 2

1.1. Catalyst Preparation 2

1.2. Catalyst Characterization 3

1.2.1. Methods 3

1.2.2. *Operando* XAS 6

1.3. Catalytic Evaluation 7

1.4. Evaluation of Mass and Heat Transfer Limitations 9

1.5. Computational Methods 10

2. Supplementary Tables 12

3. Supplementary Figures 39

4. Supplementary References 65

1. Supplementary Methods

1.1. Catalyst Preparation

The nitrogen-doped carbon support (NC) was prepared in a two-step synthesis,[1] consisting of the oxidative polymerization of aniline and a subsequent carbonization step. Aniline (50 mmol, Acros, 99.5%) was dissolved in deionized water (40 cm^3, pH 0.4; adjusted by hydrochloric acid, 1.25 M, Sigma Aldrich, >37%), cooled to 277 K, and subsequently added to a pre-cooled solution (277 K) of ammonium persulfate (50 mmol, Acros, 98%) in deionized water (20 cm^3). After 5 min of vigorous stirring, the mixture was kept at room temperature for 24 h to complete the polymerization process. The formed polyaniline was thoroughly washed with deionized water for neutralization, dried under vacuum at 393 K for 12 h, and treated at 1073 K in N_2. The obtained NC was ground and sieved (particle size 0.4-0.6 mm). Commercial activated (AC, Norit ROX 0.8) and non-activated (C, Gun Ei Chemical Ind.) carbons were ground and sieved into 0.4-0.6 mm particles.

All metal-based catalysts were prepared *via* an incipient wetness impregnation method with a nominal metal loading of 1 wt.%. The metal precursors, H_2PtCl_6 (ABCR, 99.9%, 40.0 wt.% Pt), $HAuCl_4 \cdot xH_2O$ (ABCR, 99.9%, 49.5 wt.% Au), and $RuCl_3 \cdot xH_2O$ (ABCR, 99.9%, 40.0 wt.% Ru), were dissolved in aqua regia or water (1.5 cm^3 g^{-1}) and the obtained solutions were added dropwise, under magnetic stirring, to the carbon carriers. Subsequently, all samples were dried at 333 K for 12 h. The respective catalysts were obtained *via* thermal activation (T_a, heating rate = 5 K min^{-1}, hold time = 12 h, static air if T_a < 673 K or N_2 if T_a > 673 K) and denoted as **M_X/support-solvent-T_a** (M = Pt, Au or Ru; X = single atoms (SA) or nanoparticles (NP), support = NC, AC, or C; solvent = water (w) or aqua regia (a); T_a = 473-1073 K; for example: Pt_{SA}/AC-w-473, **Supplementary Table S1**). Furthermore, a chloride-free Pt SAC was prepared by following an analogous procedure. In detail, the metal precursor $K_2Pt(CN)_4$ (Sigma Aldrich, 98%, 51.7 wt.% Pt) was dissolved in water·(1.5 cm^3 g^{-1}) and the obtained solution was added dropwise, under magnetic stirring, to the AC carrier. Subsequently, the sample was dried at 333 K for 12 h. The respective catalyst was obtained *via* thermal activation (T_a = 473 K, heating rate = 5 K min^{-1}, hold time = 12 h, static air) and denoted as $Pt(CN)_{SA}$/AC-w-473. Finally, to probe the effect of employing aqua regia as a solvent on the catalytic properties of the carbon support, the latter was first impregnated with aqua regia, dried at 473 K for 12 h, and then employed as a carrier for the

impregnation with an aqueous solution of H_2PtCl_6, as described above. The obtained catalyst was denoted as Pt_{SA}/(AC-a-473)-w-473.

1.2. Catalyst Characterization

1.2.1. Methods

Multiple characterization techniques were employed to assess the structural properties of the catalysts, as summarized in **Supplementary Table S2.**

Powder X-ray diffraction (XRD) was measured using a PANalytical X'Pert PRO-MPD diffractometer with Cu-Kα radiation (λ = 1.54060 Å). The data was recorded in the 10-70° 2θ range with an angular step size of 0.017° and a counting time of 0.26 s per step (**Supplementary Figure S1**).

Scanning transmission electron micrographs (STEM) with a high-angle annular dark-field (HAADF) detector were acquired on an aberration-corrected HD2700CS (Hitachi) microscope operated at 200 kV (**Supplementary Figure S2**). Samples were prepared by dipping the copper grid supporting a holey carbon foil in a suspension of the solid in ethanol and drying in air.

Temperature-programmed desorption analyses of acetylene were performed in a Micromeritics Autochem II 2920 analyzer equipped with a thermal conductivity detector (TCD) and a Pfeiffer Vacuum OmniStar GSD 320 O mass spectrometer (C_2H_2-TPD-MS). The samples (0.1 g) were loaded into a U-shaped quartz micro-reactor, pre-dried (T = 533 K, heating rate = 10 K min^{-1}, hold time 20 min, F_T = 20 cm^3 min^{-1}, flowing He), cooled to 303 K, and subsequently saturated with a flow of the respective probe molecule (T = 303 K, hold time 20 min, F_T = 20 cm^3 min^{-1}). After purging (T = 303 K, hold time 20 min, F_T = 20 cm^3 min^{-1}, flowing He), the desorption was initiated by increasing the temperature (T = 533 K, heating rate = 5 K min^{-1}, flowing He) while monitoring the desorbed products by mass spectrometry.

X-ray photoelectron spectra (XPS) were acquired on a Physical Electronics Quantum 2000 instrument using monochromatic Al-Kα radiation, generated from an electron beam operated at 15 kV, and equipped with a hemispherical capacitor electron-energy analyzer. The samples were analyzed at an electron take-off angle of 45° and a constant analyzer pass energy of 46.95 eV with a spectra resolution step width of 0.2 eV. The spectrometer was calibrated for the Au $4f_{7/2}$ signal at 84.0 ± 0.1 eV. The Pt $4f$,

O1*s*, and Cl 2*p* spectra were fitted after Shirley background subtraction. The selected peak positions are based on literature reported data.[2-4] The XPS analyses presented in the study providing insights into the interaction of acetylene with the carbon support were conducted on catalysts that were exposed to the relevant reactive environments, as indicated by the sample code, in the laboratory set-up (*vide infra*).

Continuous wave (CW) electron paramagnetic resonance (EPR) spectroscopy experiments were conducted on a Bruker Elexsys E500 spectrometer operating at X-band frequencies, using an ER4102ST microwave resonator and equipped with an Oxford helium (ESR900) cryostat. The CW EPR spectra were acquired at 10 K with the spectrometer settings provided in **Supplementary Table S3**. The measured g-factors were offset-corrected against a known standard (*i.e.*, free radical 1,1-diphenyl-2picrylhydrazyl). Furthermore, 2D hyperfine sublevel correlation (HYSCORE) EPR spectroscopy was performed at 10 K on a Bruker EleXsys E580 spectrometer using a split ring resonator (ER4118X-MS3) equipped with an Oxford helium (CF935P) cryostat and operating at a frequency of approximately 9.4 GHz. The measurements were conducted by applying a $\pi/2$-τ-$\pi/2$-t_1-π-t_2-$\pi/2$-τ echo pulse sequence with $\pi/2$ pulse length of 24 ns and an inversion π pulse length of 16 ns with time delays, τ, of 128 and 224 ns to avoid blind-spot artifacts. The 2D HYSCORE time-domain data were recorded by measuring the echo amplitude as a function of dimensions t_1 and t_2, incremented in steps of 8 ns. The pulse sequence was repeated in an 8-step phase cycling procedure to avoid undesired echoes. The obtained spectra were then processed by (*i*) subtracting the background decay using a polynomial function, (*ii*) zero filling to 1024 points, (*iii*) tapering using a Hamming window, and by (*iv*) Fourier transforming the data in both t_1 and t_2 dimensions. The CW and 2D HYSCORE spectra were simulated using EasySpin routines in the MATLAB software. The isotropic and anisotropic contributions to the hyperfine coupling of coke radicals and ^{195}Pt (I = 1/2, 34% isotopic abundance) nuclei could be resolved by signal fitting. The former is determined by Fermi coupling constant a_{iso} and it is related to the spin density in Pt *s* orbitals. The latter can be ascribed to either dipole-dipole interaction through space or to spin density in Pt *d* or *p* orbitals. For an axial dipolar coupling tensor, dipole-dipole interaction is described by a single parameter, T. Assuming exclusively through-space interaction and the point-dipole approximation, the distance, r, between coke deposits and the metal sites can be estimated from T by Eq. 1,[5]

$$r = \sqrt[3]{\frac{\gamma_e \gamma_n \mu_0 h}{16\pi^3 T}} \quad \text{Eq. 1}$$

where γ_e and γ_n are the electron and nuclear gyromagnetic ratios, respectively, μ_0 is the magnetic permeability of vacuum, and *h* is Planck's constant. The spin density in Pt *s* orbitals, ρ_s, is estimated from the isotropic hyperfine constant, a_0, of 34410 MHz,[6] by Eq. 2,

$$\rho_s = \frac{a_{\text{iso}}}{a_0} \quad \text{Eq. 2}$$

The uniaxial hyperfine constant of ^{195}Pt is 1474 MHz and the angular factor for *d* orbitals is 2/7, leading to a *d*-orbital hyperfine constant, $b_{0,d}$, of 421.1 MHz.[6] Assuming negligible through-space coupling, the spin density in Pt *d* orbitals was estimated by Eq. 3,

$$\rho_d = \frac{T}{b_{0,d}} \quad \text{Eq. 3}$$

Nitrogen sorption isotherms of as-prepared and used catalysts were measured at 77 K in a Micromeritics 3Flex instrument, after degassing of the solids at 423 K for 12 h (**Supplementary Tables S4**).

X-ray absorption spectroscopy (XAS) measurements were carried out at the SuperXAS beamline of the Swiss Light Source.[7] The incident photon beam provided by a 2.9 T superbend magnet was selected by a Si(111) channel-cut Quick-EXAFS monochromator.[8] The rejection of higher harmonics and collimation were achieved with Rh-coated (for the Pt L_3, Au L_3 edges) and a Pt-coating (for the Ru *K* edge) mirror, respectively, at 2.9 mrad. The area of sample illuminated by the X-ray beam was 0.5 mm×0.2 mm with focusing achieved with toroidal double focusing mirror. The beamline was calibrated using the respective metal foils. The area of sample illuminated by the X-ray beam was 0.5 mm×0.2 mm. All spectra were recorded in transmission mode at room temperature. The extended X-ray absorption fine structure (EXAFS) spectra were acquired with a 1 Hz frequency (0.5 s per spectrum). The resulting raw data were processed using the ProQEXAFS software package.[9] The EXAFS spectra were analyzed using the Demeter software package.[10] The amplitude reduction factors were refined from fitting to the corresponding metal foils. The evolution of the oxidation and coordination state of the metal components was monitored by *operando* XAS (*vide infra,*

Supplementary Figures S25, S26a-c) while selected tests (**Supplementary Figures S26b, S26c**) were replicated in the laboratory to investigate both chemical state alterations of carbon surface species and coke formation, respectively evaluated by *ex situ* XPS and EPR analyses. These studies, in combination with C_2H_2-TPD-MS analyses (*vide supra*) as well as kinetic and computational investigations (*vide infra*), enabled the resolution of the roles of the metal and carbon components in fulfilling the acetylene hydrochlorination catalytic cycle (**Supplementary Tables S5-S24, Supplementary Figures S3-S24**). The multivariate curve resolution (MCR) analysis of the XAS spectra acquired *operando* was performed using pyMCR, employing constraints for unity concentration sum and non-negativity in spectral components and concentrations.[11] The MCR refinements were completed until convergence with percentage lack of fit as the loss function. Initialization of the spectral components was performed using the SIMPLISMA algorithm.[12]

1.2.2. *Operando* XAS

Pt L_3, Au L_3, and Ru K edge quick scanning XAS were employed to probe the transient state of Pt, Au, and Ru active sites both during the synthetic process and under reactive environments relevant to acetylene hydrochlorination. Provided the corrosiveness of HCl, an experimental set-up, shown in **Supplementary Figure S25,** was designed to ensure the equipment and personnel safety. For this purpose, the set-up integrated (*i*) a small (1 L) low-concentration HCl gas bottle (1 vol.% in He), (*ii*) a custom-made ventilated fume hood to accommodate all potential sources of HCl leaks (*i.e.*, HCl gas bottle, pressure reducer, mass-flow controller), (*iii*) the equipment of the HCl mass-flow controller with an electronic shut-off valve, (*iv*) two HCl -sensors (Dräger, PointGard 2100) connected to the control box that would automatically and immediately trigger closure of HCl supply in case HCl leak detection, and (*v*) HCl traps, comprised of a 2 M NaOH solution, connected to the outlet stream.

All catalysts were tested at atmospheric pressure in a continuous-flow capillary reactor (2.8 mm internal diameter). An overview of the catalyst monitoring conditions by *operando* XAS is provided in **Supplementary Figure S26**. To monitor the thermal activation of metal species deposited on the carbon supports *via* incipient wetness impregnation, denoted as **M_X/support-solvent** (M = Pt; X = single atoms (SA) or nanoparticles (NP), support = NC, AC, or C; solvent = water (w) or aqua regia (a);

T_a = 473-1073 K; for example: Pt_{SA}/AC-w), the catalysts were heated, while collecting XAS scans, under He flow to the desired temperature, which was maintained until no more changes in the spectral features were detected, as represented in **Supplementary Figure S26a**.

To probe and evaluate changes in the metal oxidation state and coordination environment, the catalysts were exposed to acetylene hydrochlorination conditions and sequential reactive environments relevant for acetylene hydrochlorination, as represented in **Supplementary Figures S26b** and **S26c**, respectively. Reaction products were evaluated by time-resolved mass spectroscopy analysis of the capillary reactor outlet stream, monitoring the relative concentration of vinyl chloride (VCM). Prior to feeding the reactant mixtures (0-0.81 vol.% C_2H_2, 0-0.90 vol.% HCl, 2.00 vol.% Ar, 96.29-97.19 vol.% He) at a total volumetric flow rate of 68 cm^3 min^{-1}, the catalysts were heated under He flow to the desired temperature (473 K). XAS scans were collected during the whole process, and different reactant mixtures were fed only when no more changes in the spectral features were detected. Catalysts heated under He to the reaction temperature (473 K) are denoted as "**-He**" (for example: Pt_{SA}/AC-w-473-He). Catalysts exposed to acetylene hydrochlorination conditions are denoted as "**-*Y*h**" (number of hours on stream, Y = 1-10; for example: Pt_{SA}/AC-w-473-1h), while catalysts exposed to a sequence of reactive environments are denoted as "**-*Z***" (reactive environment, Z = 1-6, as indicated in **Supplementary Figure S26c**; for example: Pt_{SA}/AC-w-473-1).

To probe metal-C_2H_2 interactions, selected single-atom catalysts (SACs) were exposed to a C_2H_2 feed at 303 K and heated to 473 K under C_2H_2 flow, as represented in **Supplementary Figure S26d**. XAS scans were collected during the whole process until no more changes in the spectral features were detected. The C_2H_2-exposed catalysts are denoted as "**-C_2H_2**" (for example: Pt_{SA}/AC-w-473-C_2H_2).

1.3. Catalytic Evaluation

The hydrochlorination of acetylene was evaluated at atmospheric pressure in a continuous-flow fixed-bed micro-reactor, described elsewhere.[1] The gases C_2H_2 (PanGas, purity 2.6), HCl (Air Liquide, purity 2.8, anhydrous), Ar (PanGas, purity 5.0, internal standard), and He (PanGas, purity 5.0, carrier gas), were fed using digital mass-flow controllers (Bronkhorst) to the mixing unit, equipped with a pressure indicator. A quartz micro-reactor of 10 mm internal diameter was loaded with the catalyst

(W_{cat} = 0.25 g, particle size 0.4-0.6 mm) and placed in a home-made electrical oven. A K-type thermocouple fixed in a co-axial quartz thermowell with the tip positioned in the center of the catalyst bed was used to control the temperature during the reaction. Prior to testing, the catalyst was heated in a He flow to the desired bed temperature (T_{bed} = 473 K) and allowed to stabilize for at least 15 min before the reaction mixture (40 vol.% C_2H_2, 44 vol.% HCl, and 16 vol.% Ar) was fed at a total volumetric flow of F_T = 15 $cm^3\ min^{-1}$. Reaction kinetics of acetylene hydrochlorination over the Pt_{SA}/AC-w-473 catalyst were studied in the temperature range of 443-473 K at conversion levels <20%, with a catalyst mass of W_{cat} = 0.03 g, a total flow of F_T = 10 $cm^3\ min^{-1}$, and reactant concentrations of 10-30 vol.%, balanced in He to determine the apparent activation energy (E_a) and the partial reaction order of the reactants ($n_{C_2H_2}$, n_{HCl}). Carbon-containing compounds (C_2H_2 and C_2H_3Cl) and Ar were quantified on-line *via* a gas chromatograph equipped with a GS-Carbon PLOT column coupled to a mass spectrometer (GC-MS, Agilent, GC 7890B, Agilent MSD 5977A). Since vinyl chloride (VCM) was the only product detected in all our tests, the catalytic activity is presented as the yield of VCM, Y(VCM), calculated according to Eq. 4,

$$Y(\mathrm{VCM}),\% = \frac{n_{\mathrm{VCM}}^{\mathrm{outlet}}}{n_{\mathrm{C_2H_2}}^{\mathrm{inlet}}} \cdot 100 \qquad \text{Eq. 4}$$

where $n_{\mathrm{VCM}}^{\mathrm{outlet}}$ and $n_{\mathrm{C_2H_2}}^{\mathrm{inlet}}$ denote the molar flows of VCM and C_2H_2 at the reactor outlet and inlet, respectively. The reaction rate (r) was determined according to Eq. 5,

$$r, \mathrm{mol_{C_2H_2}\ s^{-1}\ g_{cat}^{-1}} = \frac{n_{\mathrm{C_2H_2}}^{\mathrm{inlet}} - n_{\mathrm{C_2H_2}}^{\mathrm{outlet}}}{W_{\mathrm{cat}}} \qquad \text{Eq. 5}$$

Where W_{cat} denotes the catalyst mass. The error of the carbon balance, ε_{C}, determined using Eq. 6, was less than 5% in all experiments, *i.e.*, the carbon mass balance was closed at ≥95%.

$$\varepsilon_{\mathrm{C}},\% = \frac{n_{\mathrm{C_2H_2}}^{\mathrm{inlet}} - \left(n_{\mathrm{C_2H_2}}^{\mathrm{outlet}} + n_{\mathrm{VCM}}^{\mathrm{outlet}}\right)}{n_{\mathrm{C_2H_2}}^{\mathrm{inlet}}} \cdot 100 \qquad \text{Eq. 6}$$

After the tests, the reactor was quenched to room temperature in He flow and the catalyst was retrieved for further characterization.

1.4. Evaluation of Mass and Heat Transfer Limitations

The evaluation of the dimensionless moduli based on the criteria of Carberry,[13] Mears,[14] and Weisz-Prater,[15] confirmed that all the catalytic tests were performed in the absence of mass and heat transfer limitations. The Carberry criterion (*Ca*)[13] was used to evaluate external mass transfer limitations according to Eq. 7,

$$Ca = \frac{r_{\mathrm{v,obs}}}{a' \cdot k_{\mathrm{f}} \cdot c_{\mathrm{b}}} < \frac{0.05}{|n|} \qquad \text{Eq. 7}$$

where k_{f} is the mass transfer coefficient (estimated at a minimum value of 0.01 m s^{-1}), c_{b} is the bulk concentration of acetylene (17.6 mol C_2H_2 m^{-3}), n the reaction order, while $r_{\mathrm{v,obs}}$ and a' denote the reaction rate and the specific particle area, which are derived *via* Eq. 8 and Eq. 9, respectively,

$$r_{\mathrm{v,obs}} = \frac{n_{\mathrm{C_2H_2}}^{\mathrm{inlet}} - n_{\mathrm{C_2H_2}}^{\mathrm{outlet}}}{V_{\mathrm{cat}}} \qquad \text{Eq. 8}$$

$$a' = \frac{1}{L} = \frac{A_{\mathrm{p}}}{V_{\mathrm{p}}} = \frac{6}{d_{\mathrm{p}}} \qquad \text{Eq. 9}$$

To assess extra-particle temperature gradients (ΔT_{e})[13] Eq. 10 was applied,

$$\Delta T_{\mathrm{e}} = \beta_{\mathrm{e}} \cdot Ca = \frac{(-\Delta H_{\mathrm{r}}) \cdot k_{\mathrm{f}} \cdot c_{\mathrm{b}}}{h \cdot T_{\mathrm{b}}} Ca \qquad \text{Eq. 10}$$

where β_{e} denotes the external Prater number, T_{b} the temperature in the bulk material (473 K), *h* the heat transfer coefficient (estimated at a minimum value of 10 J m^{-2} s^{-1} K^{-1}), and ΔH_{r} the reaction enthalpy (99.3 kJ mol^{-1}). Internal mass transfer limitations were evaluated using the Weisz-Prater criterion (Φ),[13] according to Eq. 11,

$$\Phi = \frac{r_{\mathrm{v,obs}} \cdot L^2}{D_{\mathrm{eff}} \cdot c_{\mathrm{s}}} \left(\frac{n+1}{2} \right) < 1 \qquad \text{Eq. 11}$$

where *L* is the characteristic length (0.25 mm), c_{s} the surface concentration ($c_{\mathrm{s}} \approx c_{\mathrm{b}}$ in the absence of external mass transfer limitations), and D_{eff} the effective diffusion coefficient, which can be derived *via* Eq. 12,

$$D_{\text{eff}} = \frac{\varepsilon}{\tau} \cdot \overline{D} = \left(\frac{1}{D_{\text{C}_2\text{H}_2,\text{HCl}}} + \frac{1}{D_{\text{K}}} \right)^{-1} \qquad \text{Eq. 12}$$

where τ is the tortuosity factor (estimated at 3), ε is the particle porosity (estimated at 0.2), $D_{\text{C}_2\text{H}_2,\text{HCl}}$ is the molecular diffusion coefficient (1.96 10^{-5} m^2 s^{-1}), and D_{K} is the Knudsen diffusion coefficient, which is calculated for components i (i = HCl, C_2H_2) in a cylindrical pore according to Eq. 13,

$$D_{\text{K},i} = 97 \cdot r_{\text{pore}} \cdot \sqrt{\frac{T}{M_i}} \qquad \text{Eq. 13}$$

where r_{pore} denotes the pore radius, M_{i} the molecular weight of component i, and T the temperature (473 K). Intra-particle temperature gradients (ΔT_{i}) can be calculated using Eq. 14,[13]

$$\Delta T_{\text{i}} = \beta_{\text{i}} \cdot T_{\text{s}} = \frac{(-\Delta H_{\text{r}}) \cdot D_{\text{eff}} \cdot c_{\text{s}}}{\lambda_{\text{eff}}} \qquad \text{Eq. 14}$$

where β_{i} denotes the internal Prater number and λ_{eff} the effective thermal conductivity (estimated at 0.5 W K^{-1} min^{-1}). The results for Pt/C catalysts are given in **Supplementary Table S25**. For all catalytic tests, the Carberry criterion (Eq. 7) is fulfilled and hence no external mass transfer limitations are present. In line with this result, the external temperature difference is negligible (< 0.01 K), indicating the absence of external heat transfer limitations. In the fresh SACs, intra-particle mass transfer limitations are largely negligible, while with decreasing pore size minor diffusional disguises might develop for NC-supported SACs, which are partially compensated by the simultaneous drop in the reaction rate.[2] Only for pore sizes <0.1 nm, intra-particle mass transfer limitations become dominant. Finally, the temperature gradient within the catalyst particle is negligible for each case, indicating the absence of internal heat transfer limitations.

1.5. Computational Methods

All DFT calculations were performed using the Vienna Ab initio Simulation Package (VASP).[16,17] The generalized gradient approximation of the Perdew-Burke-Ernzerhof functional was used to obtain the exchange-correlation energies with dispersion contributions introduced via Grimme's DFT-D3 approach.[18,19] Projector augmented wave for the core and plane waves with a cut-off energy of 450 eV for the valence were chosen with spin polarization allowed when needed.[20,21] A vacuum region

between slabs of at least 12 Å and a dipole correction along the *z*-axis were employed for slab models.[22] For all investigated systems, structures were relaxed using convergence criteria of 10^{-4} and 10^{-5} eV for the ionic and electronic steps, respectively.

To describe the extensive phase space of chemical environments in AC,[2] a set of five cavities was constructed for the simulation of the AC support, comprising slabs (6×6) of graphitic carbon with mono- to tetra-coordinated configurations with epoxide and carbonyl functionalities: (*i*) monoepoxide (epo), (*ii*) diepoxide (epo_2), (*iii*) di-ketone ($keto_2$), (*iv*) tetraketone ($keto_4$), and (*v*) edge carbonyl (edge) cavities. The AC-supported Pt single-atom catalyst was modeled by placing $PtCl_x$ (x = 0–2) moieties at the center of the distinct AC cavities. Physisorbed molecules were considered to preserve ~2/3 of their gas phase entropy,[23] as translation over the carbon plane was feasible (*i.e.*, barrier lower than 0.7 eV). T_{bed} = 473 K, F_t = 10 cm^3 min^{-1}, $p(C_2H_2, HCl)$ = 0.1-0.4 bar, W_{cat} = 0.03 g, and P = 1 bar.

2. Supplementary Tables

Supplementary Table S1. Notation, metal speciation, and details on the carbon supports employed and synthesis conditions of as-prepared catalysts.

Catalyst	Metal	Metal speciation	Support	Company	Solvent	$T_a{}^a$ / K
Pt_{SA}/AC-w-473	Pt	single atoms	activated carbon	Norit	water	473
Pt_{NP}/AC-w-873	Pt	nanoparticles	activated carbon	Norit	water	873
Pt_{SA}/AC-a-473	Pt	single atoms	activated carbon	Norit	water	473
Pt_{SA}/NC-w-473	Pt	single atoms	nitrogen-doped carbon	-	aqua regia	473
Pt_{SA}/NC-w-1073	Pt	single atoms	nitrogen-doped carbon	-	water	673
Pt_{SA}/NC-a-473	Pt	single atoms	nitrogen-doped carbon	-	water	1073
Pt_{SA}/C-w-473	Pt	single atoms	non-activated carbon	Gun Ei Chemical Ind.	aqua regia	473
Au_{SA}/AC-a-473	Au	single atoms	activated carbon	Norit	aqua regia	473
Ru_{SA}/AC-a-473	Ru	single atoms	activated carbon	Norit	aqua regia	473

[a]Temperature applied during thermal activation.

Supplementary Table S2. Overview of employed methods with their acronyms and purpose.

Technique (acronym)	Purpose
X-ray diffraction (XRD)	Carbon crystallinity and metal nuclearity
N_2 sorption	Porous properties
Scanning transmission electron microscopy (STEM) with high-angle annular dark-field (HAADF) detector	Metal nuclearity and dispersion
X-ray photoelectron spectroscopy (XPS)	Surface composition and elemental speciation
X-ray absorption spectroscopy (XAS)	Metal oxidation state and coordination environment
Electron paramagnetic resonance spectroscopy (EPR)	Composition and location of coke formation
Temperature-programmed desorption of acetylene coupled to mass spectrometry (C_2H_2-TPD-MS)	Capacity and strength of catalyst interaction with acetylene
Density functional theory (DFT)	Identification of reactant binding sites and a viable reaction profile

Supplementary Table S3. Measurement parameters employed for CW EPR spectra acquisition.

Parameters	Value
Microwave frequency / GHz	9.165
Centre field / mT	330
Sweep width / mT	20
Modulation amplitude / mT	0.05
Modulation frequency / kHz	100
Microwave power / mW	0.7
Power attenuation / dB	20
Conversion time / ms	327.68
Time constant / ms	5.12

Supplementary Table S4. Porous properties of as-prepared and used catalysts.

Catalyst	V_{total}^{a} / cm^3 g^{-1}	V_{micro}^{b} / cm^3 g^{-1}	S_{BET}^{c} / m^2 g^{-1}
Pt_{SA}/AC-w-473	0.73	0.55	1470
Pt_{SA}/AC-a-473	0.61	0.46	1385
Pt_{SA}/NC-w-473	0.24	0.14	364
Pt_{SA}/NC-w-1073	0.21	0.16	379
Pt_{SA}/C-w-473	0.04	0.02	47
Au_{SA}/AC-a-473	0.57	0.52	1079
Ru_{SA}/AC-a-473	0.68	0.54	1186
Pt_{SA}/AC-w-473-12h[d]	0.43	0.35	972
Pt_{SA}/AC-a-473-12h[d]	0.26	0.20	548
Pt_{SA}/NC-w-473-12h[d]	0.04	0.00	37
Pt_{SA}/NC-w-1073-12h[d]	0.05	0.00	47
Pt_{SA}/C-w-473-12h[d]	-	-	-
Au_{SA}/AC-a-473-12h[d]	0.48	0.34	935
Ru_{SA}/AC-a-473-12h[d]	0.29	0.18	488

[a]Volume of N_2 adsorbed at $p/p_0 = 0.98$. [b]*t*-plot method. [c]BET method. [d]Catalysts after use in acetylene hydrochlorination for 12 h on stream, as indicated in the sample code.

Supplementary Table S5. Notation and description of catalysts monitored by *operando* XAS during synthesis and under reactive environments, summarized in **Supplementary Figure S26**.

Notation[a]	Description
M_X/support-solvent	Impregnated carbon with a solution of the desired metal precursor in the selected solvent prior thermal activation
M_X/support-solvent-T_a	Thermally activated catalyst
M_X/support-solvent-T_a-He	Thermally activated catalyst heated under He to the reaction temperature (*i.e.*, 473 K)
M_X/support-solvent-T_a-Yh	Thermally activated catalyst under acetylene hydrochlorination (Y = number of hours on stream)
M_X/support-solvent-T_a-Z	Thermally activated catalyst under reactive environments (Z = reactive environment, **Supplementary Figure S26c**)
M_X/support-solvent-T_a-C_2H_2	Thermally activated catalyst exposed to acetylene

[a]Metal, M = Pt, Au, Ru; metal speciation, X = single atoms (SA) or nanoparticles (NP); support = activated carbon (AC), nitrogen-doped carbon (NC), non-activated carbon (C); solvent = water (w) or aqua regia (a); activation temperature, T_a = 473-1073 K.

Supplementary Table S6. Fitting parameters derived from the Pt L_3 edge EXAFS spectra of H_2PtCl_6 impregnated on AC and NC acquired during thermal activation (473-1023 K, **Supplementary Figure S26a**).

Catalyst	Coordination	CN^a / -	σ^{2b} / Å^2	R^c / Å	R_f^d / -	S_0^{2e} / -
Pt_{SA}/AC-w^f	Pt-C/O	0.3 ± 0.2	0.006 ± 0.002	1.82 ± 0.02	0.002	0.79
	Pt-C/O	0.8 ± 0.2	0.006 ± 0.002	2.08 ± 0.02		
	Pt-Cl	4.4 ± 0.3	0.002 ± 0.002	2.32 ± 0.02		
Pt_{SA}/AC-w-473^f	Pt-C/O	0.8 ± 0.2	0.006 ± 0.002	1.83 ± 0.02	0.003	0.79
	Pt-C/O	0.1 ± 0.2	0.006 ± 0.002	2.08 ± 0.02		
	Pt-Cl	3.3 ± 0.3	0.002 ± 0.002	2.32 ± 0.02		
Pt_{NP}/AC-w-873^f	Pt-C/O	1.2 ± 0.2	0.006 ± 0.002	1.78 ± 0.02	0.005	0.79
	Pt-C/O	1.5 ± 0.2	0.006 ± 0.002	1.95 ± 0.02		
	Pt-Cl	0.4 ± 0.3	0.002 ± 0.002	2.31 ± 0.02		
	Pt-Pt	1.3 ± 0.3	0.006 ± 0.002	2.71 ± 0.02		
Pt_{SA}/NC-w^f	Pt-C/N/O	0.2 ± 0.2	0.006 ± 0.002	2.15 ± 0.02	0.001	0.79
	Pt-Cl	4.5 ± 0.3	0.002 ± 0.002	2.30 ± 0.02		
Pt_{SA}/NC-w-473^f	Pt-C/N/O	0.9 ± 0.2	0.006 ± 0.002	1.82 ± 0.02	0.001	0.79
	Pt-C/N/O	1.2 ± 0.2	0.006 ± 0.002	1.97 ± 0.02		
	Pt-Cl	2.3 ± 0.3	0.002 ± 0.002	2.30 ± 0.02		
	Pt-Cl^g	0.4 ± 0.3	0.002^h	2.94 ± 0.02		
Pt_{SA}/NC-w-1023^f	Pt-C/N/O	2.7 ± 0.2	0.006 ± 0.002	1.94 ± 0.02	0.031	0.79
	Pt-C/N/O	1.6 ± 0.2	0.006 ± 0.002	2.01 ± 0.02		

^{a}Coordination number. ^{b}Debye-Waller factor. ^{c}Coordination shell distance. ^{d}R-factor. ^{e}Amplitude reduction factor. ^{f}Sample code: impregnated catalysts, Pt_X/support-w, and thermally activated catalyst, Pt_X/support-w-T_a. Metal speciation, X = single atoms (SA) or nanoparticles (NP); support = AC, NC; activation temperature, T_a = 473-1023 K. ^{g}Second coordination shell. ^{h}Debye-Waller factor held at the refined value for the stronger Pt-Cl coordination at ~2.35 Å due to highly correlating parameters.

Supplementary Table S7. Elemental surface concentrations of selected catalysts determined by XPS.

Catalyst	C / at.%	O / at.%	Cl / at.%
Pt_{SA}/AC-w-473	96.7	3.0	0.1
Pt_{SA}/AC-a-473	93.3	5.9	0.8
Pt_{SA}/AC-w-473-1[a]	96.3	3.5	0.2
Pt_{SA}/AC-w-473-2[a]	96.6	2.6	0.8
Pt_{SA}/AC-w-473-3[a]	95.7	3.5	0.8
Pt_{SA}/AC-w-473-4[a]	96.6	2.6	0.8
Pt_{SA}/AC-w-473-5[a]	96.8	2.5	0.7
Pt_{SA}/AC-w-473-6[a]	96.8	2.7	0.5

[a]Sample code: Pt_{SA}/AC-w-473-*Z*. Reactive environment, *Z* = 1-6 (**Supplementary Figure S26c**). 1 = He; 2, 4, 6 = HCl and C_2H_2; 3 = HCl; 5 = C_2H_2.

Supplementary Table S8. Fitting parameters derived from the Pt 4*f* XPS spectra of selected catalysts.

Catalyst	Pt(IV)[a]			Pt(II)[a]		
	Position / eV	FWHM[b] / eV	Area / %	Position / eV	FWHM[b] / eV	Area / %
Pt_{SA}/AC-w-473	73.7	2.5	32	72.3	1.8	68
Pt_{SA}/AC-a-473	73.6	2.2	100	-	-	-
Pt_{SA}/AC-w-473-1[c]	73.6	2.1	57	72.6	1.8	43
Pt_{SA}/AC-w-473-2[c]	73.6	2.0	62	72.6	1.8	38
Pt_{SA}/AC-w-473-3[c]	73.6	2.0	63	72.6	1.8	37
Pt_{SA}/AC-w-473-4[c]	73.6	2.0	75	72.6	1.9	25
Pt_{SA}/AC-w-473-5[c]	73.6	2.0	59	72.6	1.8	41
Pt_{SA}/AC-w-473-6[c]	73.6	2.0	64	72.6	1.8	36

[a]Assigned based on reference values.[2] [b]FWHM: full width at half maximum. [c]Sample code: Pt_{SA}/AC-w-473-*Z*. Reactive environment, *Z* = 1-6 (**Supplementary Figure S26c**). 1 = He; 2, 4, 6 = HCl and C_2H_2; 3 = HCl; 5 = C_2H_2.

Supplementary Table S9. Fitting parameters derived from the Cl 2*p* XPS spectra of selected catalysts.

Catalyst	Cl-C[a]			Cl-Pt[a]		
	Position / eV	FWHM[b] / eV	Area / %	Position / eV	FWHM[b] / eV	Area / %
Pt_{SA}/AC-w-473	200.4	2.4	47	198.1	1.9	53
Pt_{SA}/AC-a-473	200.4	1.5	77	198.1	1.5	23
Pt_{SA}/AC-w-473-1[c]	200.5	1.6	43	198.3	1.8	57
Pt_{SA}/AC-w-473-2[c]	200.5	1.5	61	198.2	1.6	39
Pt_{SA}/AC-w-473-3[c]	200.4	1.5	57	198.3	1.6	43
Pt_{SA}/AC-w-473-4[c]	200.4	1.8	30	198.5	1.9	70
Pt_{SA}/AC-w-473-5[c]	200.5	1.5	70	198.2	1.9	30
Pt_{SA}/AC-w-473-6[c]	200.4	1.5	66	198.2	1.7	34

[a]Assigned based on reference values.[3] [b]FWHM: full width at half maximum. [c]Sample code: Pt_{SA}/AC-w-473-*Z*. Reactive environment, *Z* = 1-6 (**Supplementary Figure S26c**). 1 = He; 2, 4, 6 = HCl and C_2H_2; 3 = HCl; 5 = C_2H_2.

Supplementary Table S10. Fitting parameters derived from the O 1*s* XPS spectra of selected catalysts.

Catalyst	O-C[a]			O=C[a]		
	Position / eV	FWHM[b] / eV	Area / %	Position / eV	FWHM[b] / eV	Area / %
Pt_{SA}/AC-w-473	533.3	2.4	65	531.6	2.1	35
Pt_{SA}/AC-a-473	533.3	2.8	87	531.4	1.8	13
Pt_{SA}/AC-w-473-1[c]	533.4	2.2	47	531.7	2.1	53
Pt_{SA}/AC-w-473-2[c]	533.4	2.5	48	531.7	2.3	52
Pt_{SA}/AC-w-473-3[c]	533.4	2.4	44	531.7	2.5	56
Pt_{SA}/AC-w-473-4[c]	533.4	2.7	48	531.7	2.5	52
Pt_{SA}/AC-w-473-5[c]	533.4	2.6	54	531.7	2.5	46
Pt_{SA}/AC-w-473-6[c]	533.4	2.0	46	531.7	2.2	54

[a]Assigned based on reference values.[4] [b]FWHM: full width at half maximum. [c]Sample code: Pt_{SA}/AC-w-473-*Z*. Reactive environment, Z = 1-6 (**Supplementary Figure S26c**). 1 = He; 2, 4, 6 = HCl and C_2H_2; 3 = HCl; 5 = C_2H_2.

Supplementary Table S11. Fitting parameters derived from the Pt L_3 edge EXAFS spectra of Pt_{SA}/AC-w-473 acquired under reaction conditions (**Supplementary Figure S26b**).

Catalyst	Coordination	CN^a / -	σ^{2b} / Å^2	R^c / Å	R_f^d / -	S_0^{2e} / -
Pt_{SA}/AC-w-473-He^f	Pt-C/O	1.8± 0.2	0.005 ± 0.002	1.81 ± 0.02	0.003	0.79
	Pt-C/O	0.9 ± 0.2	0.005 ± 0.002	1.99 ± 0.02		
	Pt-Cl	2.4 ± 0.3	0.004 ± 0.002	2.30 ± 0.02		
	Pt-Cl^g	0.6 ± 0.3	0.004^h	2.86 ± 0.02		
Pt_{SA}/AC-w-473-1h^f	Pt-C/O	1.9 ± 0.2	0.005 ± 0.002	1.81 ± 0.02	0.010	0.79
	Pt-C/O	0.9 ± 0.2	0.005 ± 0.002	1.98 ± 0.02		
	Pt-Cl	2.7 ± 0.3	0.004 ± 0.002	2.30 ± 0.02		
	Pt-Cl^g	0.6 ± 0.3	0.004^h	2.89 ± 0.02		
Pt_{SA}/AC-w-473-2h^f	Pt-C/O	1.9 ± 0.2	0.005 ± 0.002	1.81 ± 0.02	0.009	0.79
	Pt-C/O	0.7 ± 0.2	0.005 ± 0.002	2.00 ± 0.02		
	Pt-Cl	2.9 ± 0.3	0.004 ± 0.002	2.30 ± 0.02		
	Pt-Cl^g	0.7 ± 0.3	0.004^h	2.89± 0.02		
Pt_{SA}/AC-w-473-3h^f	Pt-C/O	2.0 ± 0.2	0.005 ± 0.002	1.80 ± 0.02	0.010	0.79
	Pt-C/O	0.8 ± 0.2	0.005 ± 0.002	1.97 ± 0.02		
	Pt-Cl	2.8 ± 0.3	0.004 ± 0.002	2.30 ± 0.02		
	Pt-Cl^g	0.7 ± 0.3	0.004^h	2.90 ± 0.02		
Pt_{SA}/AC-w-473-4h^f	Pt-C/O	2.1 ± 0.2	0.005 ± 0.002	1.80 ± 0.02	0.011	0.79
	Pt-C/O	0.8 ± 0.2	0.005 ± 0.002	1.98 ± 0.02		
	Pt-Cl	2.8 ± 0.3	0.004 ± 0.002	2.30 ± 0.02		
	Pt-Cl^g	0.7 ± 0.3	0.004^h	2.89 ± 0.02		
Pt_{SA}/AC-w-473-5h^f	Pt-C/O	2.0 ± 0.2	0.005 ± 0.002	1.80 ± 0.02	0.007	0.79
	Pt-C/O	0.8 ± 0.2	0.005 ± 0.002	1.99 ± 0.02		
	Pt-Cl	2.7 ± 0.3	0.004 ± 0.002	2.30 ± 0.02		
	Pt-Cl^g	0.7 ± 0.3	0.004^h	2.89± 0.02		

(continues)

Catalyst	Coordination	CN[a] / -	σ^{2b} / Å^2	R^c / Å	R_f^d / -	S_0^{2e} / -
Pt_{SA}/AC-w-473-6h[f]	Pt-C/O	2.1 ± 0.2	0.005 ± 0.002	1.80 ± 0.02	0.014	0.79
	Pt-C/O	0.9 ± 0.2	0.005 ± 0.002	1.99 ± 0.02		
	Pt-Cl	2.7 ± 0.3	0.004 ± 0.002	2.30 ± 0.02		
	Pt-Cl[g]	0.8 ± 0.3	0.004[h]	2.88± 0.02		
Pt_{SA}/AC-w-473-7h[f]	Pt-C/O	2.0 ± 0.2	0.005 ± 0.002	1.80 ± 0.02	0.009	0.79
	Pt-C/O	0.9 ± 0.2	0.005 ± 0.002	1.98 ± 0.02		
	Pt-Cl	2.8 ± 0.3	0.004 ± 0.002	2.30 ± 0.02		
	Pt-Cl[g]	0.8 ± 0.3	0.004[h]	2.88± 0.02		
Pt_{SA}/AC-w-473-8h[f]	Pt-C/O	2.0 ± 0.2	0.005 ± 0.002	1.80 ± 0.02	0.012	0.79
	Pt-C/O	0.9 ± 0.2	0.005 ± 0.002	1.97 ± 0.02		
	Pt-Cl	2.8 ± 0.3	0.004 ± 0.002	2.30 ± 0.02		
	Pt-Cl[g]	0.8 ± 0.3	0.004[h]	2.90 ± 0.02		
Pt_{SA}/AC-w-473-9h[f]	Pt-C/O	2.0 ± 0.2	0.005 ± 0.002	1.79 ± 0.02	0.013	0.79
	Pt-C/O	0.9 ± 0.2	0.005 ± 0.002	1.95 ± 0.02		
	Pt-Cl	2.9 ± 0.3	0.004 ± 0.002	2.30 ± 0.02		
	Pt-Cl[g]	0.8 ± 0.3	0.004[h]	2.90 ± 0.02		
Pt_{SA}/AC-w-473-10h[f]	Pt-C/O	2.0 ± 0.2	0.005 ± 0.002	1.80 ± 0.02	0.012	0.79
	Pt-C/O	0.9 ± 0.2	0.005 ± 0.002	1.97 ± 0.02		
	Pt-Cl	2.8 ± 0.3	0.004 ± 0.002	2.30 ± 0.02		
	Pt-Cl[g]	0.7 ± 0.3	0.004[h]	2.89 ± 0.02		

[a]Coordination number. [b]Debye-Waller factor. [c]Coordination shell distance. [d]R-factor. [e]Amplitude reduction factor. [f]Sample code: catalysts under He, Pt_{SA}/AC-w-473-He, and catalysts under reaction conditions, Pt_{SA}/AC-w-473-*Y*h. Number of hours on stream, *Y* = 1-10. [g]Second coordination shell. [h]Debye-Waller factor held at the refined value for the stronger Pt-Cl coordination at ~2.35 Å due to highly correlating parameters.

Supplementary Table S12. Fitting parameters derived from the Pt L_3 edge EXAFS spectra of Pt_{SA}/AC-w-473 acquired under sequential reactive environments (**Supplementary Figure S26c**).

Catalyst	Coordination	CN[a] / -	σ^{2b} / Å^2	R^c / Å	R_f^d / -	S_0^{2e} / -
Pt_{SA}/AC-w-473-1[f]	Pt-C/O	1.7 ± 0.2	0.005 ± 0.002	1.83 ± 0.02	0.001	0.79
	Pt-C/O	1.0 ± 0.2	0.005 ± 0.002	2.00 ± 0.02		
	Pt-Cl	2.0 ± 0.3	0.004 ± 0.002	2.31 ± 0.02		
	Pt-Cl[g]	0.6 ± 0.3	0.004[h]	2.87 ± 0.02		
Pt_{SA}/AC-w-473-2[f]	Pt-C/O	1.9 ± 0.2	0.005 ± 0.002	1.84 ± 0.02	0.001	0.79
	Pt-C/O	0.9 ± 0.2	0.005 ± 0.002	2.01 ± 0.02		
	Pt-Cl	2.4 ± 0.3	0.004 ± 0.002	2.31 ± 0.02		
	Pt-Cl[g]	0.7 ± 0.3	0.004[h]	2.87 ± 0.02		
Pt_{SA}/AC-w-473-3[f]	Pt-C/O	1.7 ± 0.2	0.005 ± 0.002	1.83 ± 0.02	0.002	0.79
	Pt-C/O	0.8 ± 0.2	0.005 ± 0.002	1.97 ± 0.02		
	Pt-Cl	2.6 ± 0.3	0.004 ± 0.002	2.30 ± 0.02		
	Pt-Cl[g]	0.7 ± 0.3	0.004[h]	2.88 ± 0.02		
Pt_{SA}/AC-w-473-4[f]	Pt-C/O	1.9 ± 0.2	0.005 ± 0.002	1.83 ± 0.02	0.002	0.79
	Pt-C/O	0.8 ± 0.2	0.005 ± 0.002	1.99 ± 0.02		
	Pt-Cl	2.5 ± 0.3	0.004 ± 0.002	2.31 ± 0.02		
	Pt-Cl[g]	0.7 ± 0.3	0.004[h]	2.88 ± 0.02		
Pt_{SA}/AC-w-473-5[f]	Pt-C/O	1.3 ± 0.2	0.005 ± 0.002	1.86 ± 0.02	0.017	0.79
	Pt-C/O	1.6 ± 0.2	0.005 ± 0.002	2.03 ± 0.02		
	Pt-Cl[g]	0.9 ± 0.3	0.004 ± 0.002	2.34 ± 0.02		
Pt_{SA}/AC-w-473-6[f]	Pt-C/O	1.4 ± 0.2	0.005 ± 0.002	1.83 ± 0.02	0.002	0.79
	Pt-C/O	1.4 ± 0.2	0.005 ± 0.002	2.01 ± 0.02		
	Pt-Cl	1.6 ± 0.3	0.004 ± 0.002	2.31 ± 0.02		
	Pt-Cl[g]	0.4 ± 0.3	0.004[h]	2.85 ± 0.02		

[a]Coordination number. [b]Debye-Waller factor. [c]Coordination shell distance. [d]R-factor. [e]Amplitude reduction factor. [f]Sample code: Pt_{SA}/AC-w-473-*Z*. Reactive environment Z = 1-6. 1 = He; 2, 4, 6 = HCl and C_2H_2; 3 = HCl; 5 = C_2H_2. [g]Second coordination shell. [h]Debye-Waller factor held at the refined value for the stronger Pt-Cl coordination at ~2.35 Å due to highly correlating parameters.

Supplementary Table S13. Fitting parameters derived from the Au L_3 edge EXAFS spectra of Au_{SA}/AC-a-473 acquired under sequential reactive environments (**Supplementary Figure S26c**).

Catalyst	Coordination	CN^a / -	σ^{2b} / Å^2	R^c / Å	R_f^d / -	S_0^{2e} / -
Au_{SA}/AC-a-473-1^f	Au-C/O	0.2 ± 0.2	0.005 ± 0.002	1.88 ± 0.02	0.007	0.85
	Au-Cl	1.8 ± 0.3	0.005 ± 0.002	2.26 ± 0.02		
	Au-Au	1.3 ± 0.3	0.008 ± 0.002	2.85 ± 0.02		
Au_{SA}/AC-a-473-2^f	Au-C/O	0.1 ± 0.2	0.005 ± 0.002	1.88 ± 0.02	0.009	0.85
	Au-Cl	1.3 ± 0.3	0.005 ± 0.002	2.25 ± 0.02		
	Au-Au	2.7 ± 0.3	0.008 ± 0.002	2.85 ± 0.02		
Au_{SA}/AC-a-473-3^f	Au-C/O	0.1 ± 0.2	0.005 ± 0.002	1.88 ± 0.02	0.010	0.85
	Au-Cl	1.3 ± 0.3	0.005 ± 0.002	2.25 ± 0.02		
	Au-Au	2.7 ± 0.3	0.008 ± 0.002	2.84 ± 0.02		
Au_{SA}/AC-a-473-4^f	Au-Cl	1.3 ± 0.3	0.005 ± 0.002	2.25 ± 0.02	0.010	0.85
	Au-Au	2.8 ± 0.3	0.008 ± 0.002	2.85 ± 0.02		
Au_{SA}/AC-a-473-5^f	Au-C/O	0.2 ± 0.2	0.005 ± 0.002	1.85 ± 0.02	0.029	0.85
	Au-Cl	0.4 ± 0.3	0.005 ± 0.002	2.26 ± 0.02		
	Au-Au	2.8 ± 0.3	0.008 ± 0.002	2.85 ± 0.02		
Au_{SA}/AC-a-473-6^f	Au-C/O	1.6 ± 0.3	0.005 ± 0.002	2.72 ± 0.02		
	Au-C/O	0.1 ± 0.2	0.005 ± 0.002	1.87 ± 0.02	0.014	0.85
	Au-Cl	1.0 ± 0.3	0.005 ± 0.002	2.25 ± 0.02		
	Au-Au	3.5 ± 0.3	0.008 ± 0.002	2.84 ± 0.02		

^{a}Coordination number. ^{b}Debye-Waller factor. ^{c}Coordination shell distance. ^{d}R-factor. ^{e}Amplitude reduction factor. ^{f}Sample code: Au_{SA}/AC-a-473-Z. Reactive environment Z = 1-6. 1 = He; 2, 4, 6 = HCl and C_2H_2; 3 = HCl; 5 = C_2H_2.

Supplementary Table S14. Fitting parameters derived from the Ru *K* edge EXAFS spectra of Ru_{SA}/AC-a-473 acquired under sequential reactive environments (**Supplementary Figure S26c**).

Catalyst	Coordination	CN^a / -	σ^{2b} / $Å^2$	R^c / Å	R_f^d / -	S_0^{2e} / -
Ru_{SA}/AC-a-473-1^f	Ru-C/O	2.9 ± 0.2	0.002 ± 0.002	1.78 ± 0.02	0.016	0.75
	Ru-C/O	2.1 ± 0.2	0.002 ± 0.002	2.09 ± 0.02		
	Ru-Cl	2.3 ± 0.3	0.004 ± 0.002	2.38 ± 0.02		
	Ru-Ru	0.1 ± 0.3	0.002 ± 0.002	2.65 ± 0.02		
Ru_{SA}/AC-a-473-2^f	Ru-C/O	3.1 ± 0.2	0.002 ± 0.002	1.79 ± 0.02	0.012	0.75
	Ru-C/O	1.9 ± 0.2	0.002 ± 0.002	2.16 ± 0.02		
	Ru-Cl	3.2 ± 0.3	0.004 ± 0.002	2.43 ± 0.02		
	Ru-Ru	0.2 ± 0.3	0.002 ± 0.002	2.65 ± 0.02		
Ru_{SA}/AC-a-473-3^f	Ru-C/O	2.3 ± 0.2	0.002 ± 0.002	1.82 ± 0.02	0.015	0.75
	Ru-C/O	2.5 ± 0.2	0.002 ± 0.002	2.16 ± 0.02		
	Ru-Cl	3.2 ± 0.3	0.004 ± 0.002	2.44 ± 0.02		
	Ru-Ru	0.1 ± 0.3	0.002 ± 0.002	2.65 ± 0.02		
Ru_{SA}/AC-a-473-4^f	Ru-C/O	2.2 ± 0.2	0.002 ± 0.002	1.82 ± 0.02	0.014	0.75
	Ru-C/O	1.9 ± 0.2	0.002 ± 0.002	2.15 ± 0.02		
	Ru-Cl	3.3 ± 0.3	0.004 ± 0.002	2.44 ± 0.02		
	Ru-Ru	0.1 ± 0.3	0.002 ± 0.002	2.65 ± 0.02		
Ru_{SA}/AC-a-473-5^f	Ru-C/O	2.0 ± 0.2	0.002 ± 0.002	1.80 ± 0.02	0.009	0.75
	Ru-C/O	2.5 ± 0.2	0.002 ± 0.002	2.15 ± 0.02		
	Ru-Cl	1.5 ± 0.3	0.004 ± 0.002	2.42 ± 0.02		
	Ru-Ru	0.2 ± 0.3	0.002 ± 0.002	2.65 ± 0.02		
Ru_{SA}/AC-a-473-6^f	Ru-C/O	2.3 ± 0.2	0.002 ± 0.002	1.82 ± 0.02	0.001	0.75
	Ru-C/O	2.3 ± 0.2	0.002 ± 0.002	2.15 ± 0.02		
	Ru-Cl	2.7 ± 0.3	0.004 ± 0.002	2.44 ± 0.02		
	Ru-Ru	0.1 ± 0.3	0.002 ± 0.002	2.65 ± 0.02		

[a]Coordination number. [b]Debye-Waller factor. [c]Coordination shell distance. [d]R-factor. [e]Amplitude reduction factor. [f]Sample code: Ru_{SA}/AC-a-473-*Z*. Reactive environment *Z* = 1-6. 1 = He; 2, 4, 6 = HCl and C_2H_2; 3 = HCl; 5 = C_2H_2.

Supplementary Table S15. Fitting parameters derived from the Pt L_3 edge EXAFS spectra of Pt_{SA}/NC-w-473 acquired under sequential reactive environments (**Supplementary Figure S26c**).

Catalyst	Coordination	CN^a / -	σ^{2b} / Å^2	R^c / Å	R_f^d / -	S_0^{2e} / -
Pt_{SA}/NC-w-473-1^f	Pt-C/N/O	1.1 ± 0.2	0.006 ± 0.002	1.82 ± 0.02	0.004	0.79
	Pt-C/N/O	1.9 ± 0.2	0.006 ± 0.002	1.98 ± 0.02		
	Pt-Cl	2.1 ± 0.3	0.004 ± 0.002	2.30 ± 0.02		
	Pt-Cl^g	0.6 ± 0.3	0.004^h	2.93 ± 0.02		
Pt_{SA}/NC-w-473-2^f	Pt-C/N/O	1.0 ± 0.2	0.006 ± 0.002	1.76 ± 0.02	0.005	0.79
	Pt-C/N/O	1.4 ± 0.2	0.006 ± 0.002	1.96 ± 0.02		
	Pt-Cl	2.6 ± 0.3	0.004 ± 0.002	2.30 ± 0.02		
	Pt-Cl^g	0.2 ± 0.3	0.004^h	2.93 ± 0.02		
Pt_{SA}/NC-w-473-3^f	Pt-C/N/O	1.3 ± 0.2	0.006 ± 0.002	1.86 ± 0.02	0.001	0.79
	Pt-C/N/O	1.4 ± 0.2	0.006 ± 0.002	2.01 ± 0.02		
	Pt-Cl	2.4 ± 0.3	0.004 ± 0.002	2.30 ± 0.02		
	Pt-Cl^g	0.4 ± 0.3	0.004^h	2.95 ± 0.02		
Pt_{SA}/NC-w-473-4^f	Pt-C/N/O	1.3 ± 0.2	0.006 ± 0.002	1.86 ± 0.02	0.005	0.79
	Pt-C/N/O	1.4 ± 0.2	0.006 ± 0.002	2.01 ± 0.02		
	Pt-Cl	2.4 ± 0.3	0.004 ± 0.002	2.30 ± 0.02		
	Pt-Cl^g	0.4 ± 0.3	0.004^h	2.95 ± 0.02		

(continues)

Catalyst	Coordination	CN[a] / -	σ^{2b} / Å^2	R^c / Å	$R_f{}^d$ / -	$S_0{}^{2e}$ / -
Pt_{SA}/NC-w-473-5[f]	Pt-C/N/O	1.3 ± 0.2	0.006 ± 0.002	1.86 ± 0.02	0.010	0.79
	Pt-C/N/O	1.6 ± 0.2	0.006 ± 0.002	2.01 ± 0.02		
	Pt-Cl	1.8 ± 0.3	0.004 ± 0.002	2.30 ± 0.02		
	Pt-Pt	0.2 ± 0.3	0.006 ± 0.002	2.83 ± 0.02		
	Pt-Cl[g]	0.3 ± 0.3	0.004[h]	2.92 ± 0.02		
Pt_{SA}/NC-w-473-6[f]	Pt-C/N/O	1.4 ± 0.2	0.006 ± 0.002	1.86 ± 0.02	0.005	0.79
	Pt-C/N/O	1.4 ± 0.2	0.006 ± 0.002	2.01 ± 0.02		
	Pt-Cl	2.2 ± 0.3	0.004 ± 0.002	2.30 ± 0.02		
	Pt-Cl[g]	0.6 ± 0.3	0.004[h]	2.92 ± 0.02		

[a]Coordination number. [b]Debye-Waller factor. [c]Coordination shell distance. [d]R-factor. [e]Amplitude reduction factor. [f]Sample code: Pt_{SA}/NC-w-473-*Z*. Reactive environment Z = 1-6. 1 = He; 2, 4, 6 = HCl and C_2H_2; 3 = HCl; 5 = C_2H_2. [g]Second coordination shell. [h]Debye-Waller factor held at the refined value for the stronger Pt-Cl coordination at ~2.35 Å due to highly correlating parameters.

Supplementary Table S16. Fitting parameters derived from the Pt L_3 edge EXAFS spectra of Pt_{SA}/C-w-473 acquired under sequential reactive environments (**Supplementary Figure S26c**).

Catalyst	Coordination	CN^a / -	σ^{2b} / Å^2	R^c / Å	R_f^d / -	S_0^{2e} / -
Pt_{SA}/C-w-473-1^f	Pt-C/O	0.4 ± 0.2	0.006 ± 0.002	1.95 ± 0.02	0.003	0.79
	Pt-C/O	0.7 ± 0.2	0.006 ± 0.002	2.12 ± 0.02		
	Pt-Cl	3.2 ± 0.3	0.004 ± 0.002	2.31 ± 0.02		
	Pt-Cl^g	0.4 ± 0.3	0.004^h	2.85 ± 0.02		
Pt_{SA}/C-w-473-2^f	Pt-C/O	0.7 ± 0.2	0.006 ± 0.002	1.90 ± 0.02	0.002	0.79
	Pt-C/O	0.7 ± 0.2	0.006 ± 0.002	2.15 ± 0.02		
	Pt-Cl	2.6 ± 0.3	0.004 ± 0.002	2.31 ± 0.02		
	Pt-Cl^g	0.5 ± 0.3	0.004^h	2.85 ± 0.02		
Pt_{SA}/C-w-473-3^f	Pt-C/O	0.5 ± 0.2	0.006 ± 0.002	1.82 ± 0.02	0.004	0.79
	Pt-C/O	0.4 ± 0.2	0.006 ± 0.002	1.95 ± 0.02		
	Pt-Cl	1.8 ± 0.3	0.004 ± 0.002	2.30 ± 0.02		
	Pt-Pt	4.5 ± 0.3	0.006 ± 0.002	2.75 ± 0.02		
Pt_{SA}/C-w-473-4^f	Pt-C/O	0.4 ± 0.2	0.006 ± 0.002	1.78 ± 0.02	0.003	0.79
	Pt-C/O	0.5 ± 0.2	0.006 ± 0.002	1.95 ± 0.02		
	Pt-Cl	1.0 ± 0.3	0.004 ± 0.002	2.30 ± 0.02		
	Pt-Pt	5.9 ± 0.3	0.006 ± 0.002	2.75 ± 0.02		
Pt_{SA}/C-w-473-5^f	Pt-C/O	0.8 ± 0.2	0.006 ± 0.002	1.95 ± 0.02	0.016	0.79
	Pt-C/O	0.2 ± 0.2	0.006 ± 0.002	2.15 ± 0.02		
	Pt-Cl	0.8 ± 0.3	0.004 ± 0.002	2.30 ± 0.02		
	Pt-Pt	6.2 ± 0.3	0.006 ± 0.002	2.76 ± 0.02		
Pt_{SA}/C-w-473-6^f	Pt-C/O	0.4 ± 0.2	0.006 ± 0.002	1.78 ± 0.02	0.003	0.79
	Pt-C/O	0.6 ± 0.2	0.006 ± 0.002	1.97 ± 0.02		
	Pt-Cl	0.6 ± 0.3	0.004 ± 0.002	2.30 ± 0.02		
	Pt-Pt	6.6 ± 0.3	0.006 ± 0.002	2.75 ± 0.02		

^{a}Coordination number. ^{b}Debye-Waller factor. ^{c}Coordination shell distance. ^{d}R-factor. ^{e}Amplitude reduction factor. ^{f}Sample code: Pt_{SA}/C-w-473-*Z*. Reactive environment Z = 1-6. 1 = He; 2, 4, 6 = HCl and C_2H_2; 3 = HCl; 5 = C_2H_2. ^{g}Second coordination shell. ^{h}Debye-Waller factor held at the refined value for the stronger Pt-Cl coordination at ~2.35 Å due to highly correlating parameters.

Supplementary Table S17. Fitting parameters derived from the Pt L_3 edge EXAFS spectra of Pt_{SA}/AC-a-473 acquired under sequential environments (**Supplementary Figure S26c**).

Catalyst	Coordination	CN^a / -	σ^{2b} / Å^2	R^c / Å	R_f^d / -	S_0^{2e} / -
Pt_{SA}/AC-a-473-1^f	Pt-C/O	2.0 ± 0.2	0.006 ± 0.002	1.81 ± 0.02	0.001	0.79
	Pt-C/O	0.5 ± 0.2	0.006 ± 0.002	2.01 ± 0.02		
	Pt-Cl	3.4 ± 0.3	0.004 ± 0.002	2.32 ± 0.02		
	Pt-Cl^g	0.9 ± 0.3	0.004^h	2.91 ± 0.02		
Pt_{SA}/AC-a-473-2^f	Pt-C/O	2.1 ± 0.2	0.006 ± 0.002	1.83 ± 0.02	0.001	0.79
	Pt-C/O	0.7 ± 0.2	0.006 ± 0.002	2.05 ± 0.02		
	Pt-Cl	2.9 ± 0.3	0.004 ± 0.002	2.31 ± 0.02		
	Pt-Cl^g	1.0 ± 0.3	0.004^h	2.90 ± 0.02		
Pt_{SA}/AC-a-473-3^f	Pt-C/O	2.0 ± 0.2	0.006 ± 0.002	1.84 ± 0.02	0.001	0.79
	Pt-C/O	1.0 ± 0.2	0.006 ± 0.002	2.05 ± 0.02		
	Pt-Cl	2.5 ± 0.3	0.004 ± 0.002	2.32 ± 0.02		
	Pt-Cl^g	0.6 ± 0.3	0.004^h	2.88 ± 0.02		
Pt_{SA}/AC-a-473-4^f	Pt-C/O	2.0 ± 0.2	0.006 ± 0.002	1.85 ± 0.02	0.002	0.79
	Pt-C/O	1.0 ± 0.2	0.006 ± 0.002	2.06 ± 0.02		
	Pt-Cl	2.4 ± 0.3	0.004 ± 0.002	2.32 ± 0.02		
	Pt-Cl^g	0.8 ± 0.3	0.004^h	2.92 ± 0.02		
Pt_{SA}/AC-a-473-5^f	Pt-C/O	1.6 ± 0.2	0.006 ± 0.002	1.86 ± 0.02	0.003	0.79
	Pt-C/O	2.0 ± 0.2	0.006 ± 0.002	2.05 ± 0.02		
	Pt-Cl	0.7 ± 0.3	0.004 ± 0.002	2.37 ± 0.02		
	Pt-Pt	0.2 ± 0.3	0.006 ± 0.002	2.71 ± 0.02		
Pt_{SA}/AC-a-473-6^f	Pt-C/O	1.8 ± 0.2	0.006 ± 0.002	1.84 ± 0.02	0.003	0.79
	Pt-C/O	1.8 ± 0.2	0.006 ± 0.002	2.04 ± 0.02		
	Pt-Cl	1.2 ± 0.3	0.004 ± 0.002	2.34 ± 0.02		
	Pt-Cl^g	0.2 ± 0.3	0.004^h	2.91 ± 0.02		

[a]Coordination number. [b]Debye-Waller factor. [c]Coordination shell distance. [d]R-factor. [e]Amplitude reduction factor. [f]Sample code: Pt_{SA}/AC-a-473-Z. Reactive environment Z = 1-6. 1 = He; 2, 4, 6 = HCl and C_2H_2; 3 = HCl; 5 = C_2H_2. [g]Second coordination shell. [h]Debye-Waller factor held at the refined value for the stronger Pt-Cl coordination at ~2.35 Å due to highly correlating parameters.

Supplementary Table S18. Fitting parameters derived from the Pt L_3 edge EXAFS spectra of Pt_{SA}(CN)/AC-w-473 acquired under reaction conditions (**Supplementary Figure S26b**).

Catalyst	Coordination	CN[a] / -	σ^{2b} / Å^2	R^c / Å	R_f^d / -	S_0^{2e} / -
Pt(CN)$_{SA}$/AC-w-473-He[f]	Pt-C/N/O	5.8 ± 0.2	0.006 ± 0.002	1.98 ± 0.02	0.008	0.79
	Pt-C/N/O	5.6 ± 0.2	0.006 ± 0.002	3.12 ± 0.02		
Pt(CN)$_{SA}$/AC-w-473-2h[f]	Pt-C/N/O	4.3 ± 0.2	0.007 ± 0.002	1.97 ± 0.02	0.002	0.79
	Pt-C/N/O	4.3 ± 0.2	0.007 ± 0.002	3.12 ± 0.02		
	Pt-Cl	0.8 ± 0.3	0.004 ± 0.002	2.35 ± 0.02		

[a]Coordination number. [b]Debye-Waller factor. [c]Coordination shell distance. [d]R-factor. [e]Amplitude reduction factor. [f]Sample code: catalyst under He, Pt(CN)$_{SA}$/AC-w-473-He, and catalysts under reaction conditions, Pt(CN)$_{SA}$/AC-*Y*h. Number of hours on stream, $Y = 2$.

Supplementary Table S19. Fitting parameters derived from the Pt L_3 edge EXAFS spectra of selected AC-supported Pt SACs acquired under C_2H_2 (**Supplementary Figure S26d**).

Catalyst	Coordination	CN^a / -	σ^{2b} / Å^2	R^c / Å	R_f^d / -	S_0^{2e} / -
Pt_{SA}/AC-w-473	Pt-C/O	0.9 ± 0.2	0.006 ± 0.002	1.82 ± 0.02	0.002	0.79
	Pt-C/O	0.2 ± 0.2	0.006 ± 0.002	2.04 ± 0.02		
	Pt-Cl	3.1 ± 0.3	0.002 ± 0.002	2.31 ± 0.02		
	Pt-Cl^g	0.7 ± 0.3	0.002^h	2.91 ± 0.02		
Pt_{SA}/AC-w-473-C_2H_2^{f}	Pt-C/O	1.0 ± 0.2	0.006 ± 0.002	1.87 ± 0.02	0.004	0.79
	Pt-C/O	2.0 ± 0.2	0.006 ± 0.002	2.04 ± 0.02		
	Pt-Cl	0.7 ± 0.3	0.002 ± 0.002	2.36 ± 0.02		
	Pt-Pt	0.5 ± 0.3	0.006 ± 0.002	2.71 ± 0.02		
Pt_{SA}/AC-a-473	Pt-C/O	1.5 ± 0.2	0.006 ± 0.002	1.82 ± 0.02	0.005	0.79
	Pt-C/O	0.2 ± 0.2	0.006 ± 0.002	2.04 ± 0.02		
	Pt-Cl	3.1 ± 0.3	0.002 ± 0.002	2.31 ± 0.02		
	Pt-Cl^g	1.0 ± 0.3	0.002^h	2.90 ± 0.02		
Pt_{SA}/AC-a-473-C_2H_2^{f}	Pt-C/O	1.2 ± 0.2	0.006 ± 0.002	1.89 ± 0.02	0.003	0.79
	Pt-C/O	1.5 ± 0.2	0.006 ± 0.002	2.05 ± 0.02		
	Pt-Cl	1.0 ± 0.3	0.002 ± 0.002	2.36 ± 0.02		
	Pt-Cl^g	0.1 ± 0.3	0.002^h	2.90 ± 0.02		

[a]Coordination number. [b]Debye-Waller factor. [c]Coordination shell distance. [d]R-factor. [e]Amplitude reduction factor. [f]Sample code: catalysts under a C_2H_2 environment, Pt_{SA}/AC-solvent-473-C_2H_2. Solvent = w or a. [g]Second coordination shell. [h]Debye-Waller factor held at the refined value for the stronger Pt-Cl coordination at ~2.35 Å due to highly correlating parameters.

Supplementary Table S20. Fitting parameters derived from the Pt L_3 edge EXAFS spectra of selected NC-supported Pt SACs acquired under C_2H_2 (**Supplementary Figure S26d**).

Catalyst	Coordination	CN^a / -	σ^{2b} / Å^2	R^c / Å	R_f^d / -	S_0^{2e} / -
Pt_{SA}/NC-w-473	Pt-C/O	0.5 ± 0.2	0.006 ± 0.002	1.90 ± 0.02	0.003	0.79
	Pt-C/O	1.5 ± 0.2	0.006 ± 0.002	2.00 ± 0.02		
	Pt-Cl	1.8 ± 0.3	0.002 ± 0.002	2.30 ± 0.02		
	Pt-Cl^g	0.4 ± 0.3	0.002^h	2.93 ± 0.02		
Pt_{SA}/NC-w-473-C_2H_2^{f}	Pt-C/O	1.0 ± 0.3	0.006 ± 0.002	1.91 ± 0.02	0.004	0.79
	Pt-C/O	1.7 ± 0.2	0.006 ± 0.002	2.01 ± 0.02		
	Pt-Cl	1.1 ± 0.3	0.002 ± 0.002	2.30 ± 0.02		
	Pt-Cl^g	0.3 ± 0.3	0.002^h	2.94 ± 0.02		
Pt_{SA}/NC-w-1073	Pt-C/N/O	1.3 ± 0.3	0.006 ± 0.002	1.80 ± 0.02	0.022	0.79
	Pt-C/N/O	3.3 ± 0.2	0.006 ± 0.002	1.95 ± 0.02		
Pt_{SA}/NC-w-1073-C_2H_2^{f}	Pt-C/N/O	1.3 ± 0.3	0.006 ± 0.002	1.79 ± 0.02	0.024	0.79
	Pt-C/N/O	3.3 ± 0.2	0.006 ± 0.002	1.95 ± 0.02		

^{a}Coordination number. ^{b}Debye-Waller factor. ^{c}Coordination shell distance. ^{d}R-factor. ^{e}Amplitude reduction factor. ^{f}Sample code: catalysts under a C_2H_2 environment, Pt_{SA}/NC-w-T_a-C_2H_2. activation temperature, T_a = 473-1073 K. ^{g}Second coordination shell. ^{h}Debye-Waller factor held at the refined value for the stronger Pt-Cl coordination at ~2.35 Å due to highly correlating parameters.

Supplementary Table S21. Adsorption energies (E_{ads}, in eV) of $PtCl_x$ ($x = 0$-3) species on distinct AC-cavities.

Cavity[a]	Metal species			
	$PtCl_3$	$PtCl_2$	PtCl	Pt
epo_2	−2.40	−3.12	−4.30	−3.73
CO (edge)	−2.00	−2.67	−3.58	−3.96
$keto_2$	−1.07	−2.02	−2.32	−3.30
$keto_4$	−2.40	−2.95	−3.64	−6.73
COOH (edge)	−1.38	−2.07	−2.63	−3.51
OH	-[b]	-[b]	-[b]	-[b]

[a]$E_{ads} = E_{ads}(PtCl_x/AC) - E(PtCl_x, gp) - E(AC)$, where $E_{ads}(PtCl_x/AC)$ is the energy of the AC-cavity with adsorbed $PtCl_x$, $E(PtCl_x, gp)$ is the energy of the isolated $PtCl_x$ fragment in the gas-phase, and $E(AC)$ is the energy of the empty AC-cavity. [b]Formation of volatile species.

Supplementary Table S22. Adsorption Gibbs free energies (G_{ads}, in eV) of acetylene over $PtCl_2$ and C/O binding sites on distinct cavities.

Cavity[a]	Binding site				
	C/O				$PtCl_2$
	Pristine AC		AC anchoring $PtCl_2$		
	5-membered ring	6-membered ring	5-membered ring	6-membered ring	
epo_2	−0.89	−0.48	-[b]	-[b]	0.87
CO (edge)	−0.69	−0.68	−0.57	−0.45	0.54
$keto_2$	0.20	−0.02	−0.14	−0.71	0.68
$keto_4$	−0.07	−0.08	−0.75	−0.45	0.74
COOH (edge)	−0.73	−0.67	−0.35	0.03	0.80

[a] $G_{ads} = G(C_2H_2^*) - G(^*) - G(C_2H_2, \text{gp})$, where $G(C_2H_2^*)$ is the Gibbs free energy of the binding site with adsorbed acetylene, $G(^*)$ is the Gibbs free energy of the binding site, and $G(C_2H_2, \text{gp})$ is the Gibbs free energy of acetylene in the gas phase. [b] Formation of volatile species.

Supplementary Table S23. Difference in the adsorption Gibbs free energy (ΔG_{ads}, in eV) of hydrogen chloride and acetylene on $PtCl_x$ (x = 0-2) species on distinct AC-cavities.

Cavity[a]	Metal species		
	$PtCl_2$	PtCl	Pt
epo_2	−0.35	0.55	1.41
CO (edge)	−0.52	−0.30	−0.41
$keto_2$	−0.35	−0.01	0.28
$keto_4$	−1.09	0.09	−0.12
COOH (edge)	-[b]	-[b]	-[b]

[a]$\Delta G_{ads} = G(HCl^*) - G(C_2H_2^*)$, where $G_{ads}(HCl^*)$ and $G_{ads}(C_2H_2^*)$ are the Gibbs free energy of the binding site with adsorbed acetylene and hydrogen chloride, respectively. [b]Hydrogen chloride adsorption not possible owing to the absence of basic sites as proton acceptors.

Supplementary Table S24. Reaction energies of acetylene hydrochlorination catalyzed over bifunctional metal-carbon sites in $PtCl_2$/keto$_4$ species.

Reaction species[a]	ΔE / eV	ΔG / eV
C_2H_2 + HCl	0.00	0.00
$H^{\#}$ + Cl* + $C_2H_2^{\#}$	−0.09	1.19
TS	0.51	1.64
Cl* + $C_2H_3^{\#}$	−0.16	0.20
$C_2H_3Cl^{\#}$	−1.24	0.97
C_2H_3Cl	−1.59	−0.95

[a]Adsorbed species over the metal sites are denoted by *, while those adsorbed on the carbon sites are denoted by #.

Supplementary Table S25. Results for mass and heat transfer limitation criteria for selected catalysts.

Catalyst	$r_{v,obs}$ / mol C_2H_2 s^{-1} m_{cat}^{-3}	r_{pore}^{a} / nm	Ca^{b} / -	ΔT_e^{c} / K	Φ^{d} / -	ΔT_i^{e} / K
Pt_{SA}/AC-w-473	3.7	1.7	0.0018	0.006	0.85	0.08
Pt_{SA}/NC-w-473	3.9	1.8	0.0019	0.007	0.91	0.10

[a]Pore radius, estimated using N_2 sorption. [b]Carberry criterion. [c]Extra-particle temperature gradient. [d]Weisz-Prater criterion. [e]Intra-particle temperature gradient.

3. Supplementary Figures

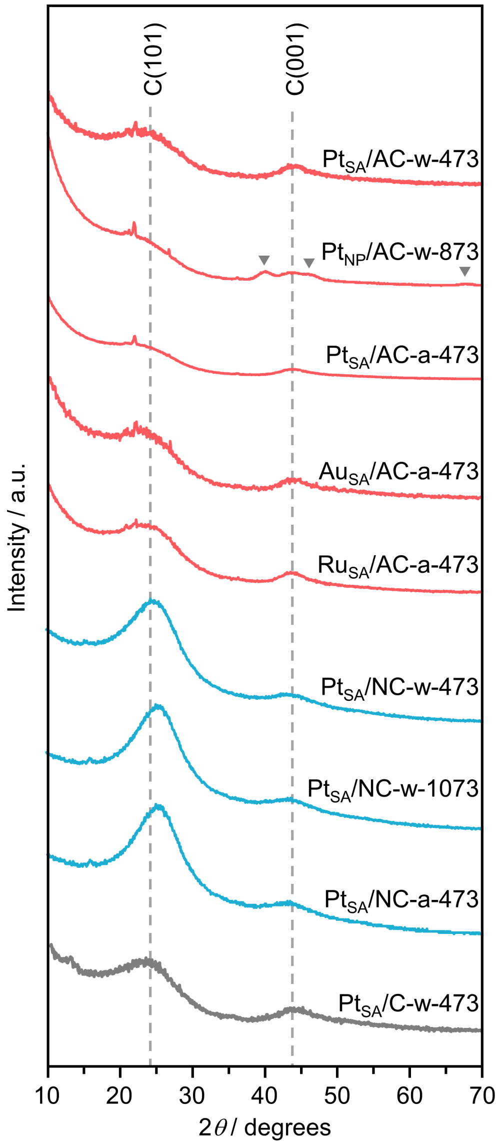

Supplementary Figure S1. XRD patterns of the as-prepared catalysts examined in this study. Diffraction peaks of carbon are indicated by the vertical gray dotted lines. Diffraction peaks of metallic platinum are detected only in Pt_{NP}/AC-w-873, as indicated by the inverted triangles. Diffraction peaks at low angles (< 24°) are attributed to incomplete activation of the commercial AC support.[24] Sample code: **M_X/support-solvent-T_a**. M = Pt, Au, or Ru; metal speciation, X = single atoms (SA) or nanoparticles (NP), support = NC, AC, or C; solvent = water (w) or aqua regia (a); activation temperature, T_a = 473-1073 K.

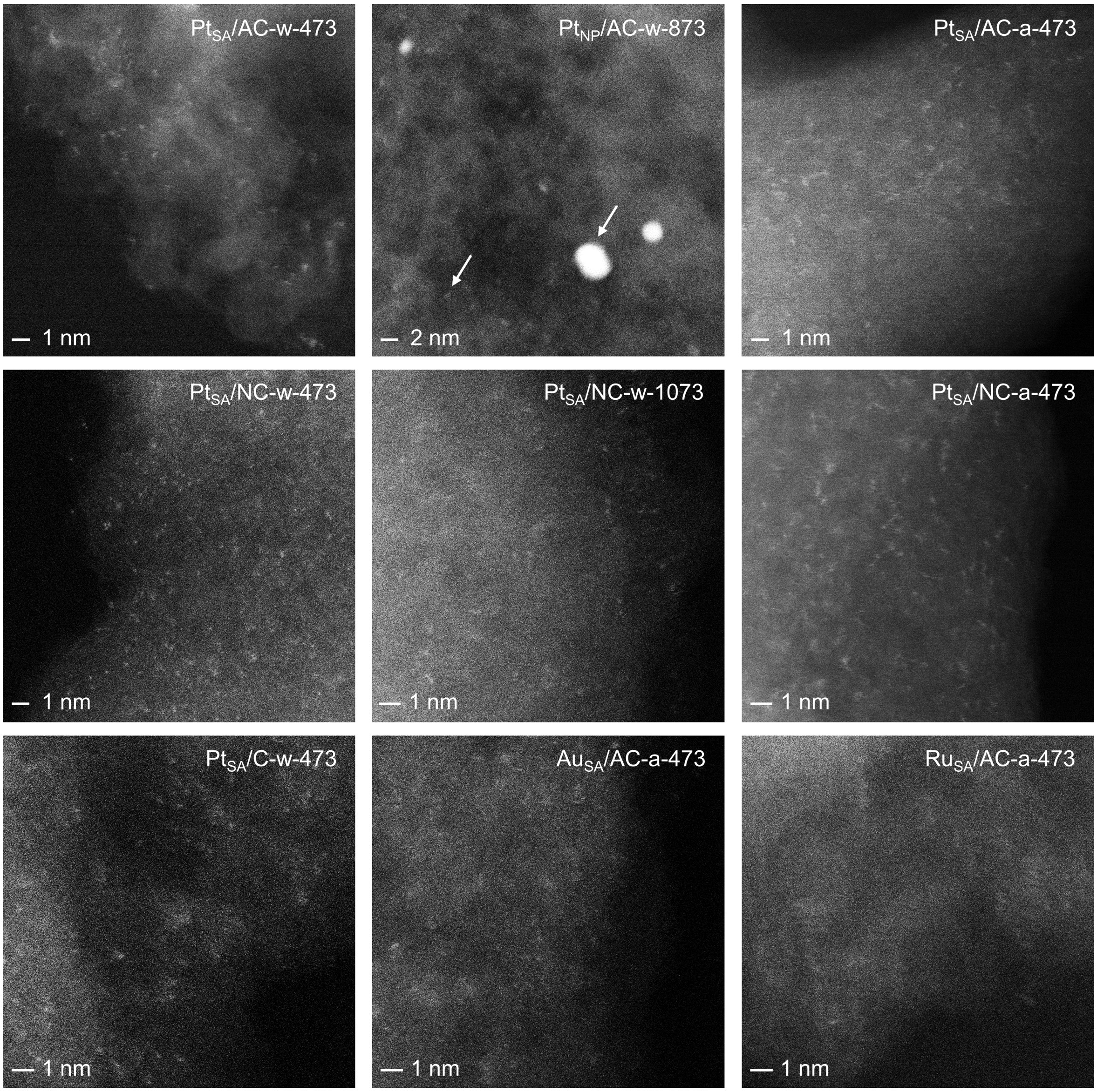

Supplementary Figure S2. HAADF-STEM images of the as-prepared catalysts examined in this study, visualizing atomic metal dispersion in all SACs and mixed metal speciation in Pt_{NP}/AC-w-873, which encompasses nanoparticles and low-nuclearity species, as indicated by the white arrows.

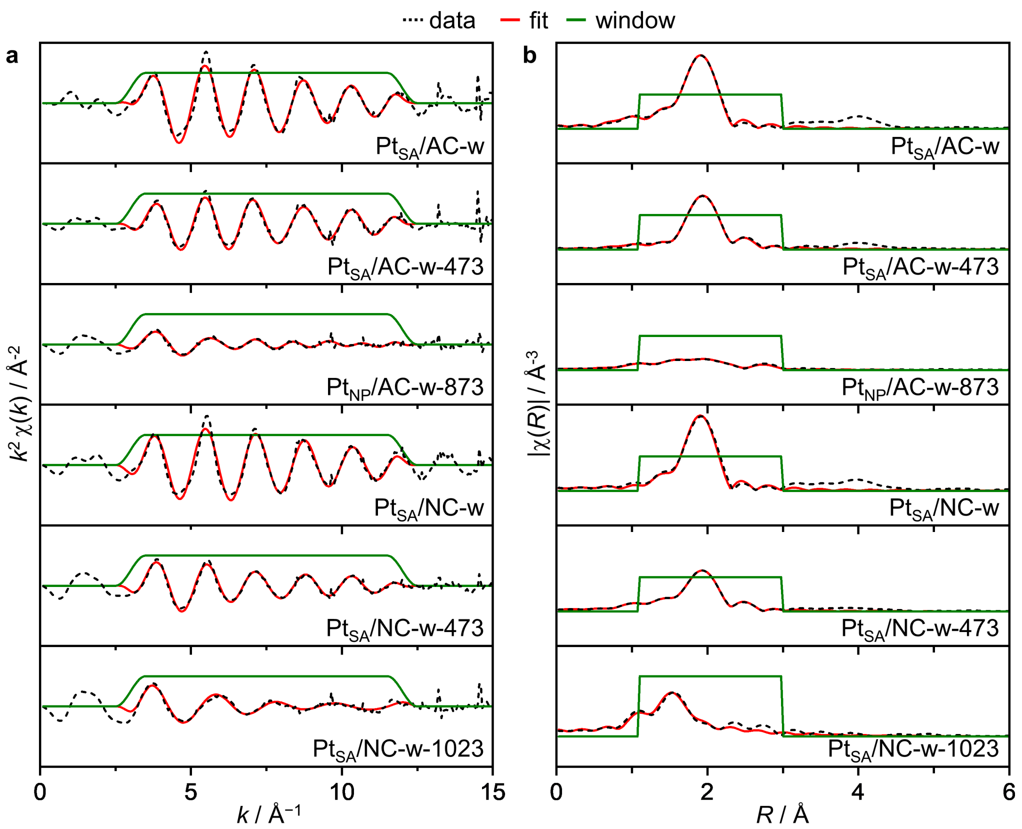

Supplementary Figure S3. a, *k* space and **b**, *R* space of experimental and fitted Pt L_3 edge EXAFS spectra of H_2PtCl_6 impregnated on AC and NC acquired during thermal activation (**Supplementary Figure S26a**). Sample code: impregnated catalysts, **Pt*X*/support-w**, and thermally activated catalyst, **Pt*X*/support-w-*T*a**. Metal speciation, X = single atoms (SA) or nanoparticles (NP); support = AC, NC; activation temperature, T_a = 473-1023 K.

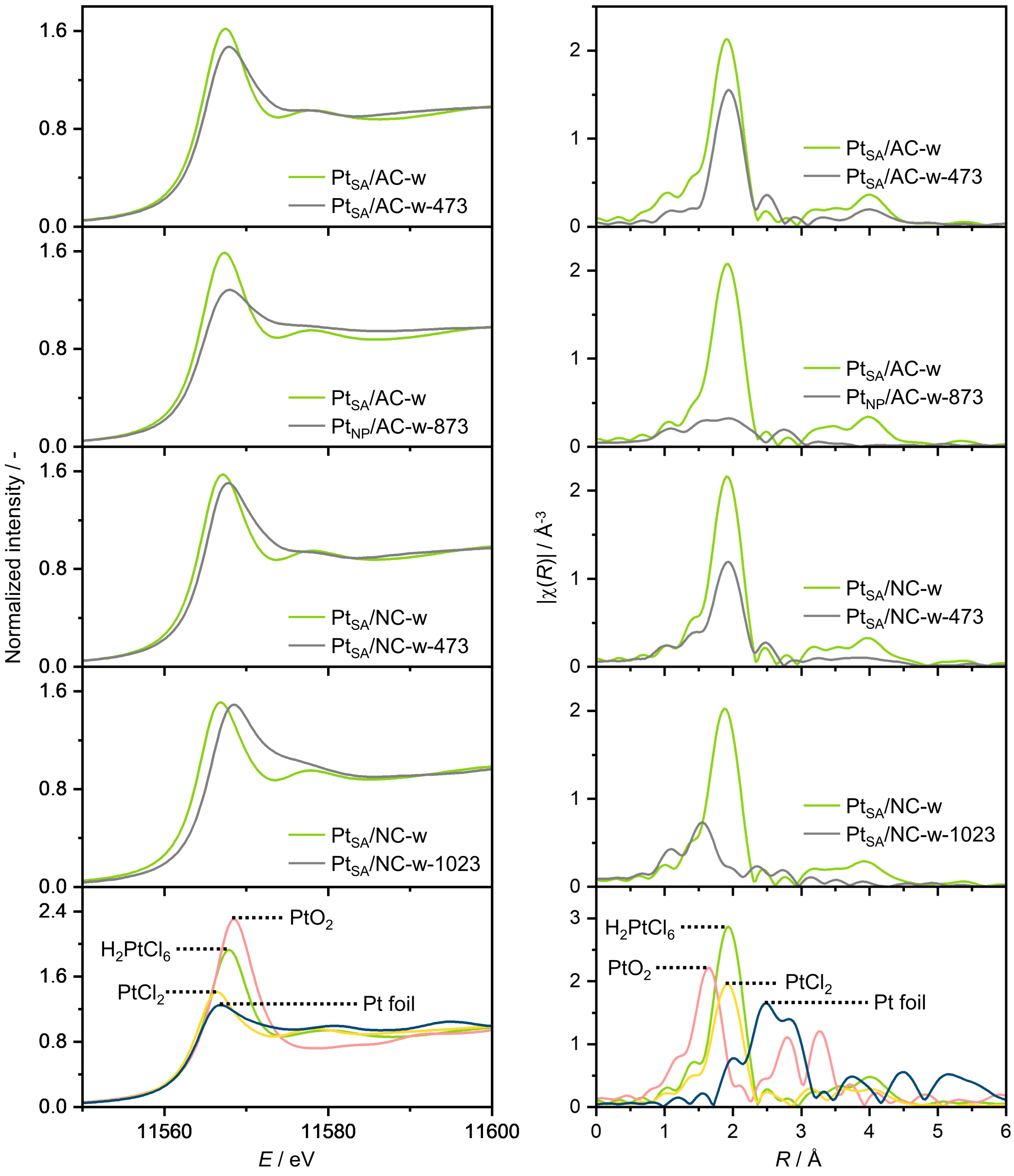

Supplementary Figure S4. Experimental Pt L_3 edge XANES (left) and EXAFS (right) spectra of H_2PtCl_6 impregnated on AC and NC acquired during thermal activation (**Supplementary Figure S26a**), together with reference spectra. Sample code: impregnated catalysts, **Pt$_X$/support-w**, and thermally activated catalyst, **Pt$_X$/support-w-T_a**. Metal speciation, X = single atoms (SA) or nanoparticles (NP); support = AC, NC; activation temperature, T_a = 473-1023 K.

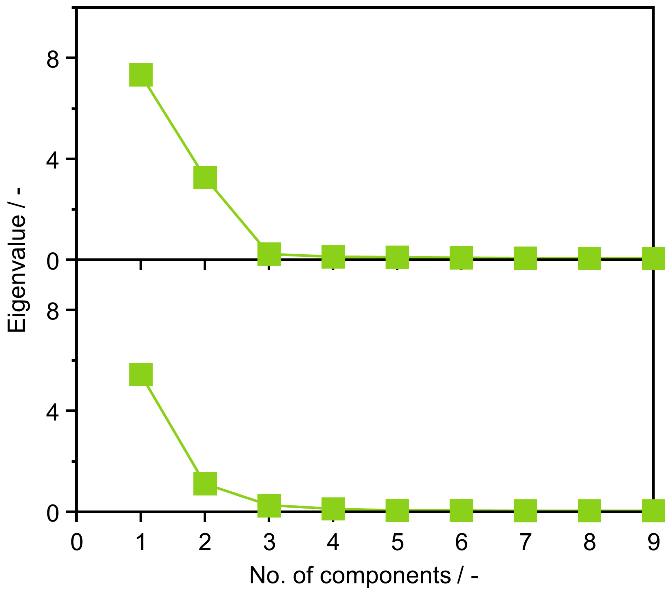

Supplementary Figure S5. Singular value decomposition analysis of Pt L_3 edge XAS spectra of H_2PtCl_6 impregnated on AC (top) and NC (bottom) during thermal activation, showing that three components are needed to minimize the eigenvalue when conducting the MCR analysis of the process. Significant reduction in the eigenvalue does not further arise from increasing the number of components.

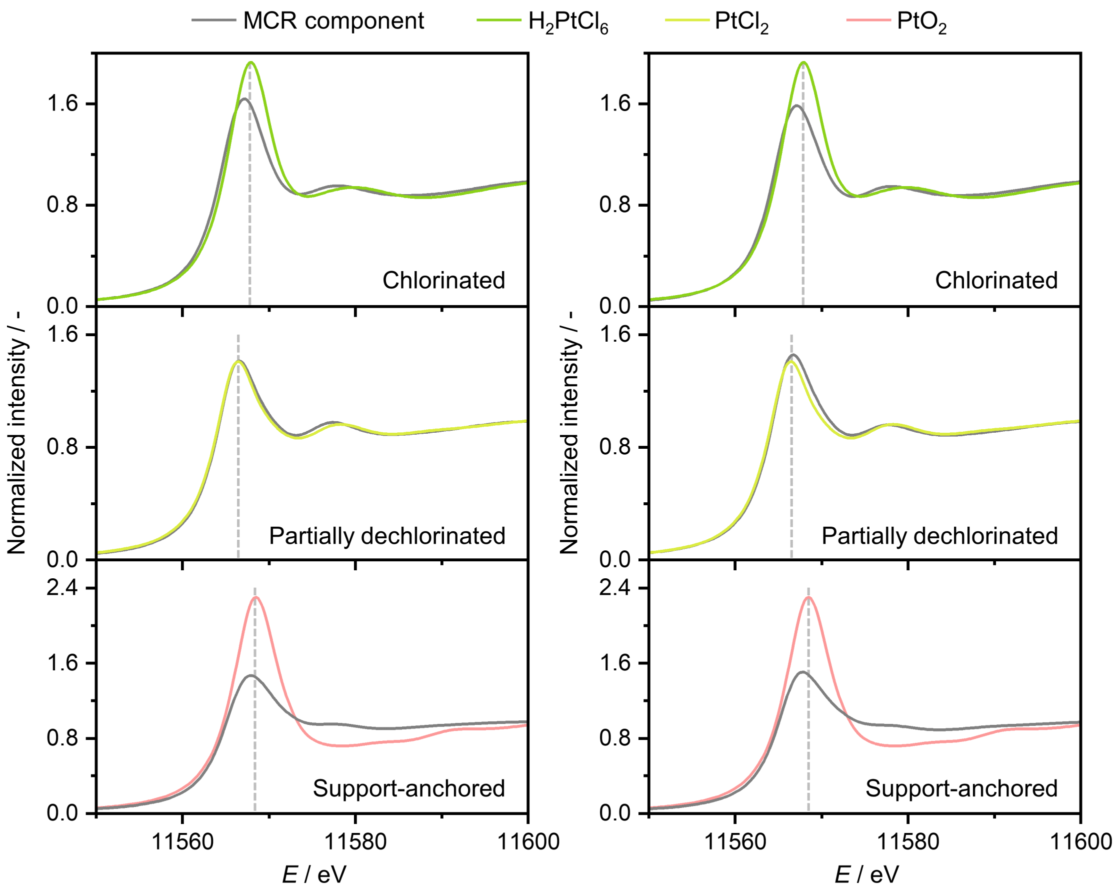

Supplementary Figure S6. Spectral components of the MCR analysis of the Pt L_3 edge XAS spectra of H_2PtCl_6 impregnated on AC (left) and NC (right) acquired during thermal activation (**Supplementary Figure S26a**), together with reference spectra. The white line intensity position of the latter ones is marked by dotted lines. The three spectral components are individually compared with the H_2PtCl_6, $PtCl_2$, and PtO_2 references, respectively reflecting the evolving "chlorinated", "partially dechlorinated", and "support-anchored" metal speciation during the thermal treatment (**Figure 2d**). The spectral components are denoted accordingly. Specifically, prominent dechlorination is observed at first, reflected in a decreasing contribution of the chlorinated component in favor of the dechlorinated one. This is followed by a shift in the white line position toward higher energy, aligning with the one of the PtO_2 reference, though the latter presents a greater intensity.

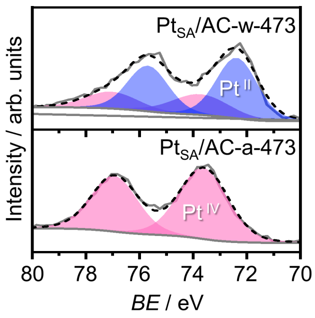

Supplementary Figure S7. Pt 4*f* XPS spectra of Pt SACs derived from impregnation of AC with H_2PtCl_6 dissolved in water and aqua regia, showing the distinct oxidation state of the resultant metal atoms.

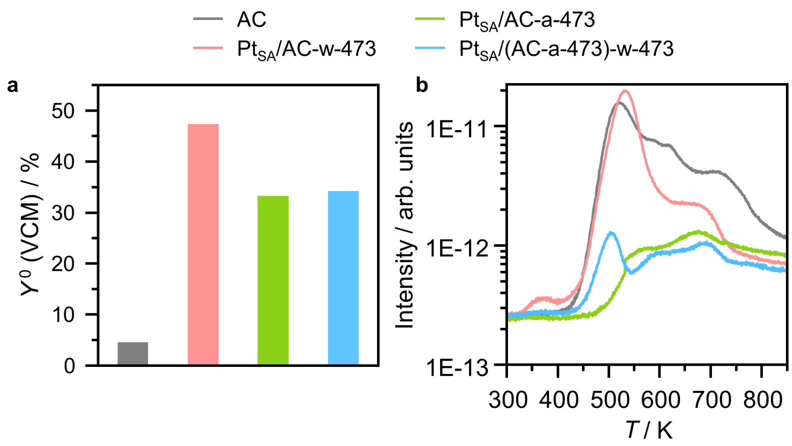

Supplementary Figure S8. a, Initial activity (expressed as VCM yield after 1 h on stream, Y^0(VCM), top) and **b**, Time-resolved product analysis in C_2H_2-TPD-MS analysis of AC-supported Pt SACs and the bare support.

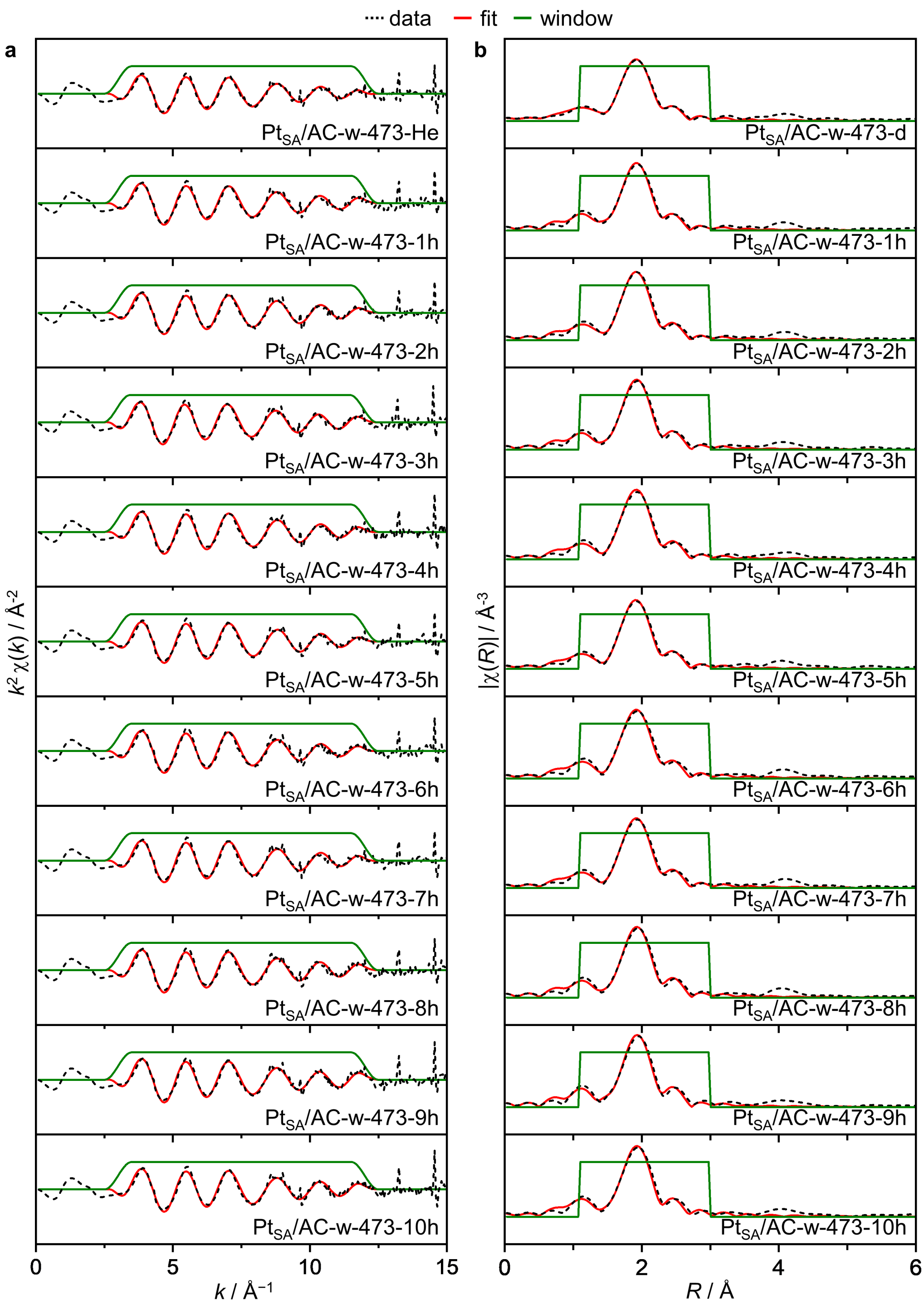

Supplementary Figure S9. a, k space and **b**, R space of experimental and fitted Pt L_3 edge EXAFS spectra of Pt_{SA}/AC-w-473 acquired under reaction conditions (**Supplementary Figure S26b**). Sample code: catalysts under He, **Pt_{SA}/AC-w-473-He**, and catalysts under reaction conditions, **Pt_{SA}/AC-w-473-*Y*h**. Number of hours on stream, Y = 1-10.

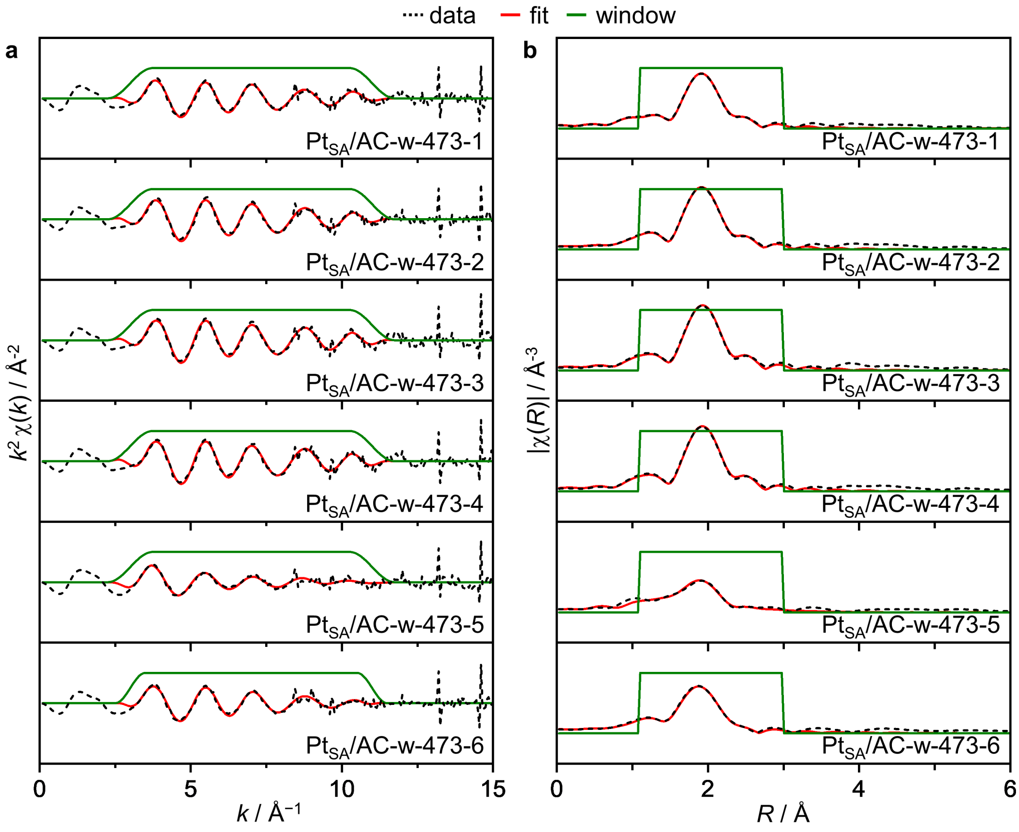

Supplementary Figure S10. a, k space and **b**, R space of experimental and fitted Pt L_3 edge EXAFS spectra of Pt_{SA}/AC-w-473 acquired under sequential reactive environments (**Supplementary Figure S26c**). Sample code: **Pt_{SA}/AC-w-473-*Z***. Reactive environment, Z = 1-6. 1 = He; 2, 4, 6 = HCl and C_2H_2; 3 = HCl; 5 = C_2H_2.

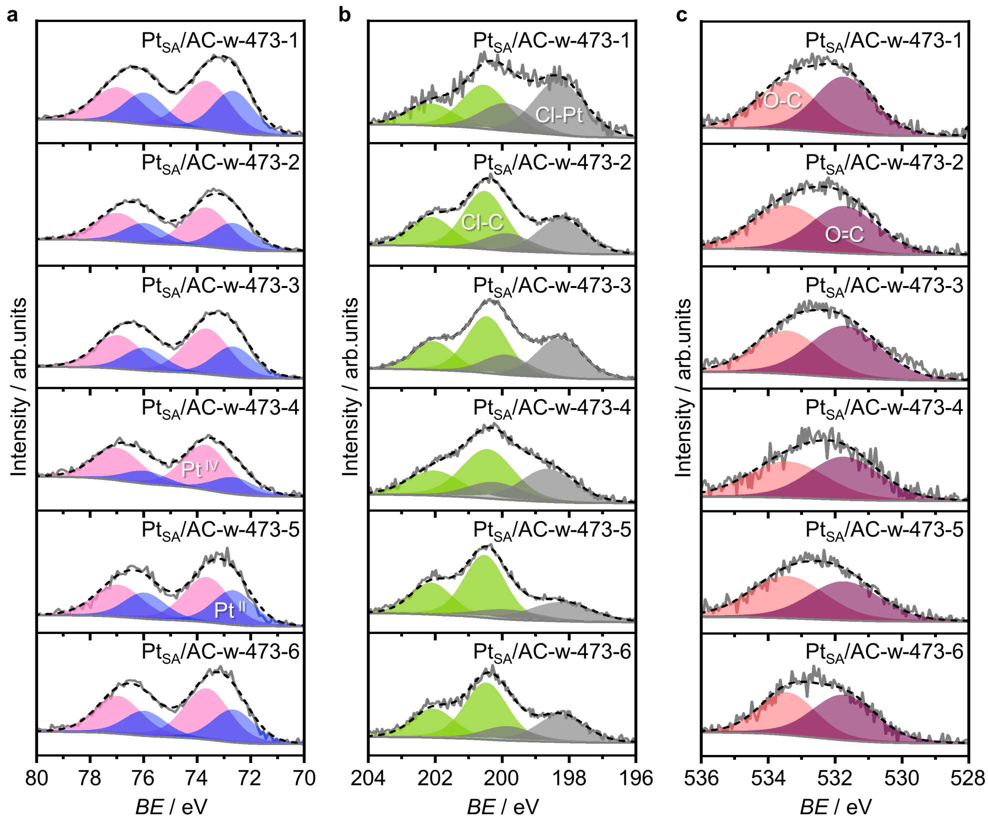

Supplementary Figure S11. a, Pt 4*f*, **b,** Cl2*p*, and **c,** O1*s* experimental and fitted XPS spectra of Pt$_{SA}$/AC-w-473 acquired after exposure to sequential reactive environments (**Supplementary Figure S26c**). Sample code: **Pt$_{SA}$/AC-w-473-*Z***. Reactive environment, *Z* = 1-6. 1 = He; 2, 4, 6 = HCl and C_2H_2; 3 = HCl; 5 = C_2H_2.

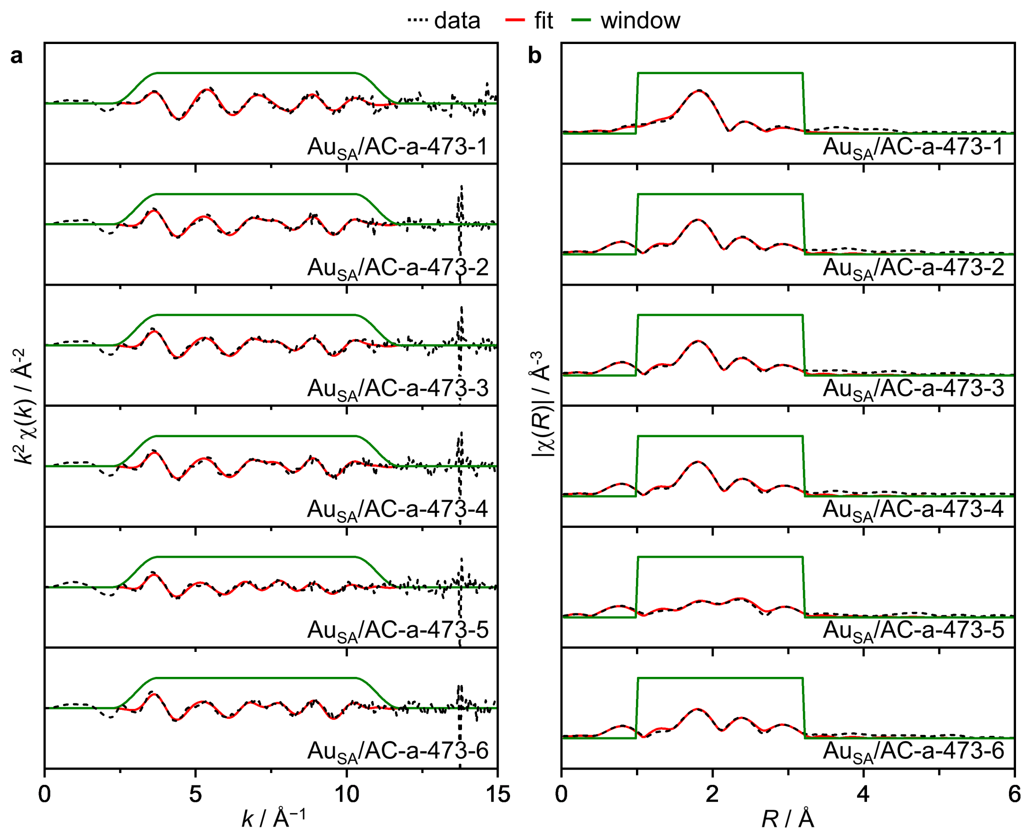

Supplementary Figure S12. a, k space and **b**, R space of experimental and fitted AuL_3 edge EXAFS spectra of Au$_{SA}$/AC-a-473 acquired under sequential reactive environments (**Supplementary Figure S26c**). Sample code: **Au$_{SA}$/AC-a-473-*Z***. Reactive environment, Z = 1-6. 1 = He; 2, 4, 6 = HCl and C_2H_2; 3 = HCl; 5 = C_2H_2.

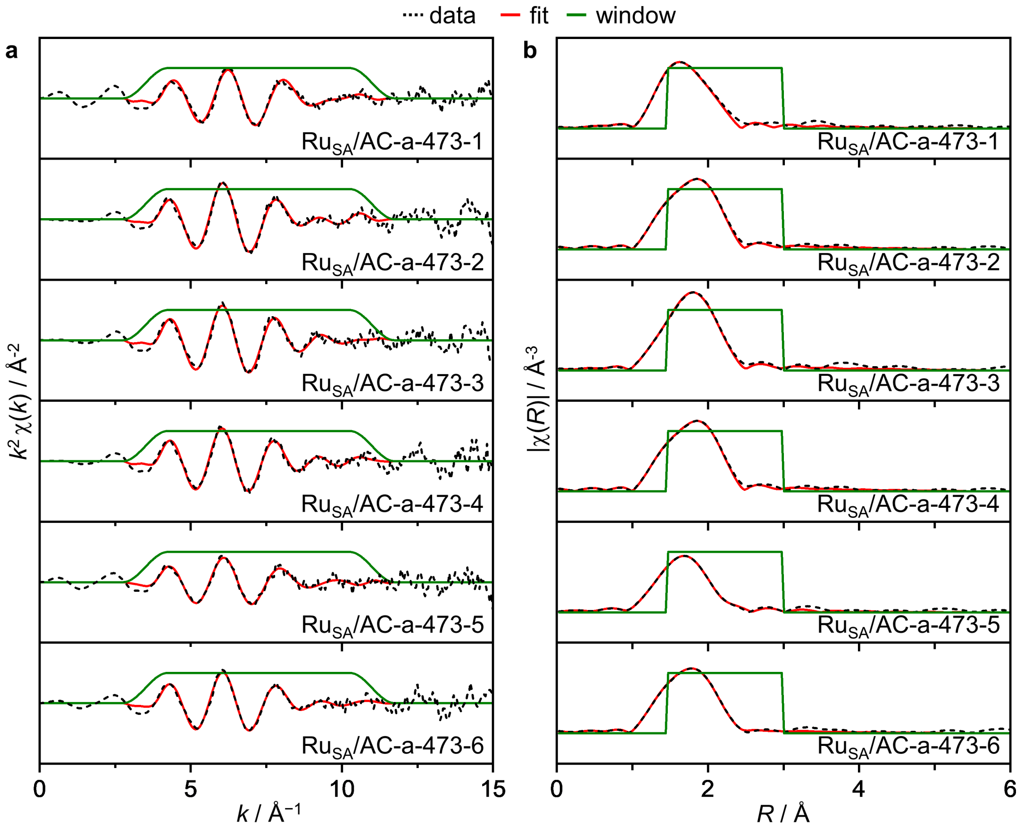

Supplementary Figure S13. a, *k* space and **b**, *R* space of experimental and fitted Ru *K* edge EXAFS spectra of Ru_{SA}/AC-a-473 acquired under sequential reactive environments (**Supplementary Figure S26c**). Sample code: **Ru_{SA}/AC-a-473-*Z***. Reactive environment, *Z* = 1-6. 1 = He; 2, 4, 6 = HCl and C_2H_2; 3 = HCl; 5 = C_2H_2.

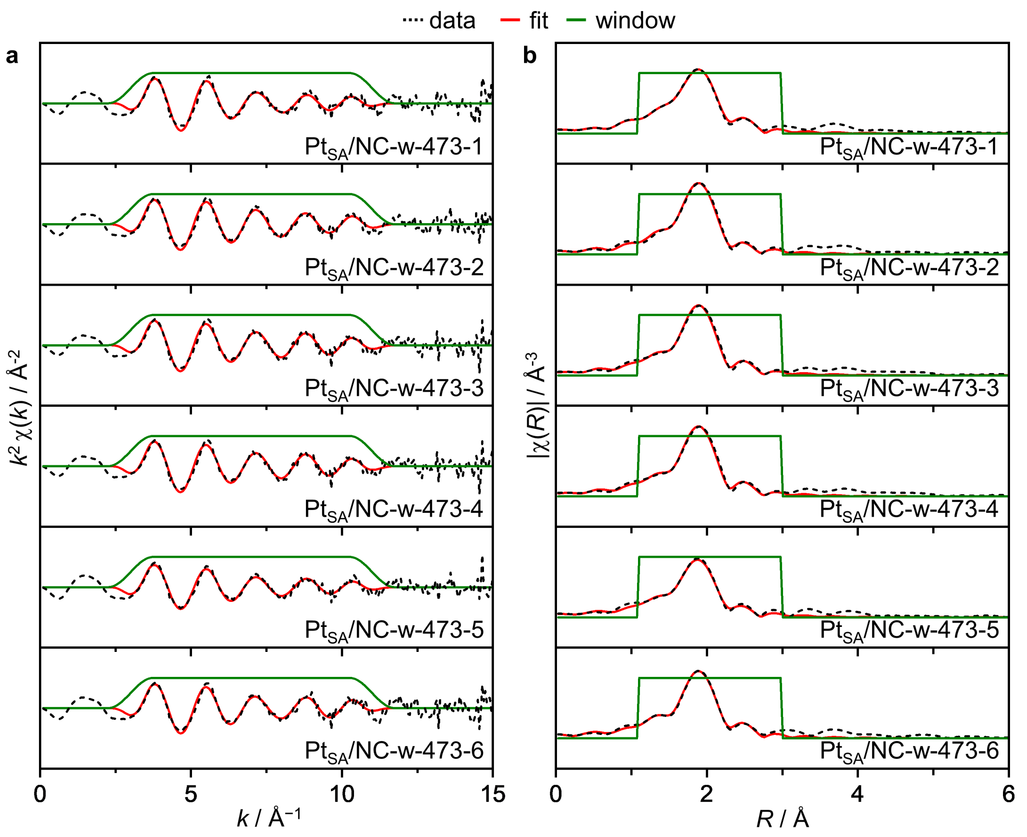

Supplementary Figure S14. a, k space and **b**, R space of experimental and fitted Pt L_3 edge EXAFS spectra of Pt$_{SA}$/NC-w-473 acquired under sequential reactive environments (**Supplementary Figure S26c**). Sample code: **Pt$_{SA}$/NC-w-473-*Z***. Reactive environment, Z = 1-6. 1 = He; 2, 4, 6 = HCl and C_2H_2; 3 = HCl; 5 = C_2H_2.

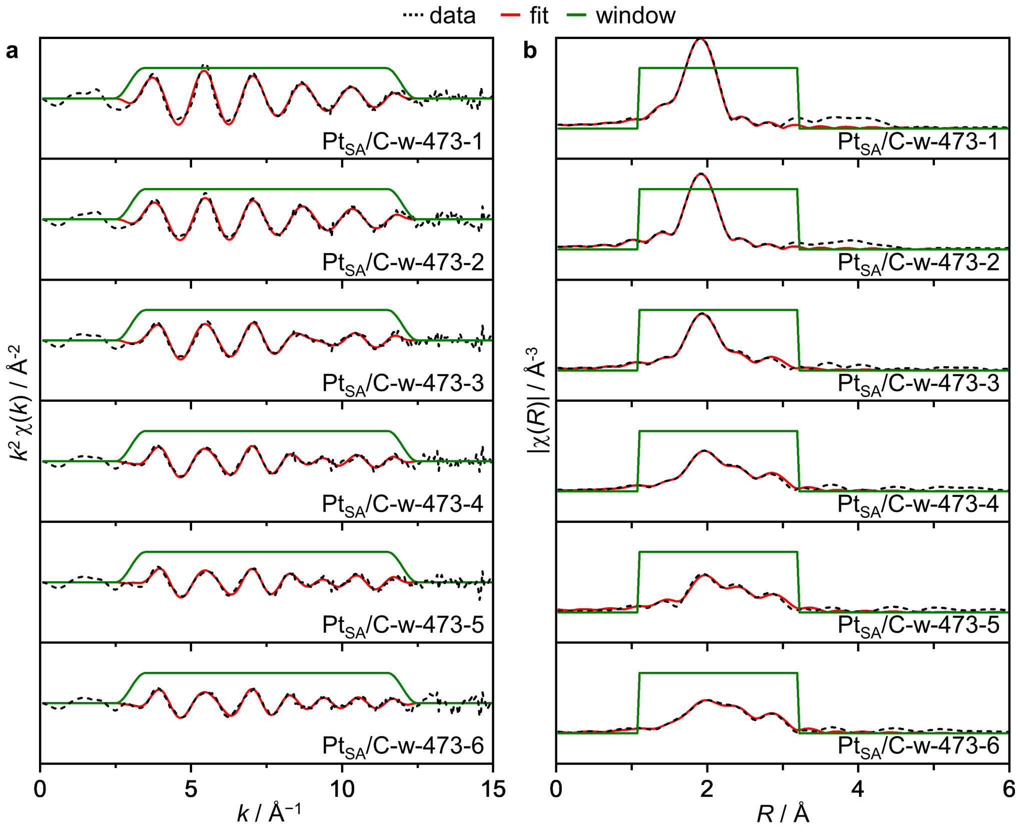

Supplementary Figure S15. a, *k* space and **b**, *R* space of experimental and fitted Pt L_3 edge EXAFS spectra of Pt$_{SA}$/C-w-473 acquired under sequential reactive environments (**Supplementary Figure S26c**). Sample code: **Pt$_{SA}$/C-w-473-*Z***. Reactive environment, *Z* = 1-6. 1 = He; 2, 4, 6 = HCl and C_2H_2; 3 = HCl; 5 = C_2H_2.

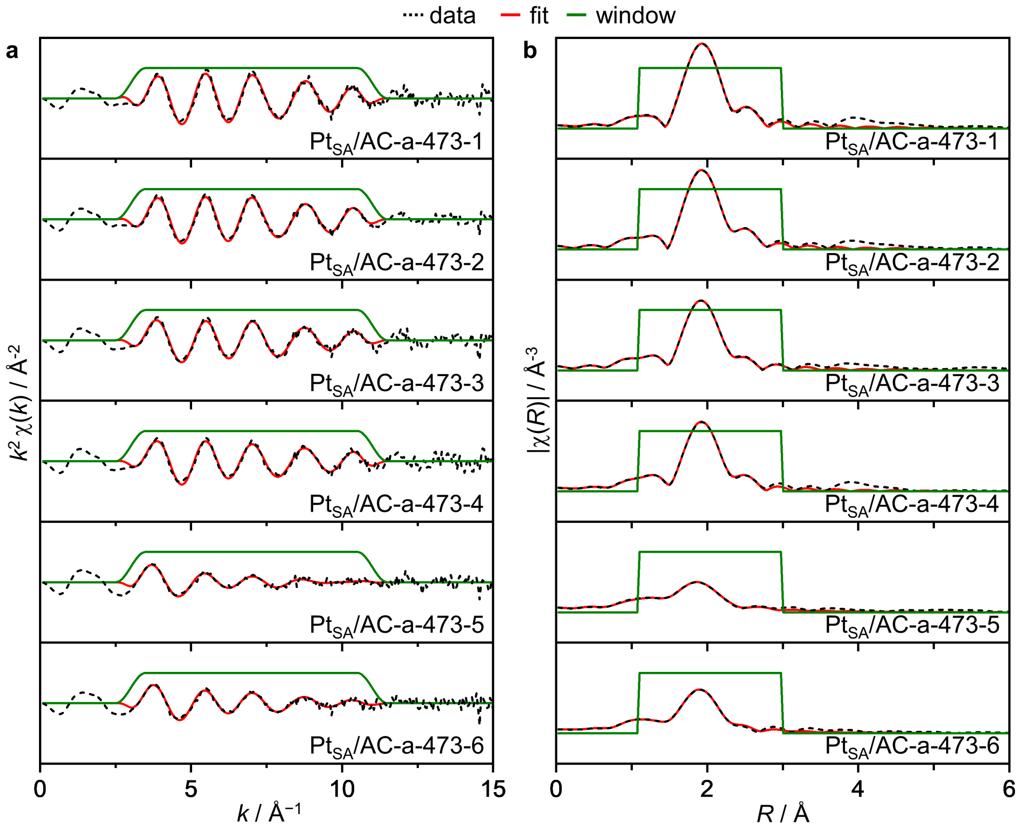

Supplementary Figure S16. a, k space and **b**, R space of experimental and fitted Pt L_3 edge EXAFS spectra of Pt_{SA}/AC-a-473 acquired under sequential reactive environments (**Supplementary Figure S26c**). Sample code: **Pt_{SA}/AC-a-473-*Z***. Reactive environment, Z = 1-6. 1 = He; 2, 4, 6 = HCl and C_2H_2; 3 = HCl; 5 = C_2H_2.

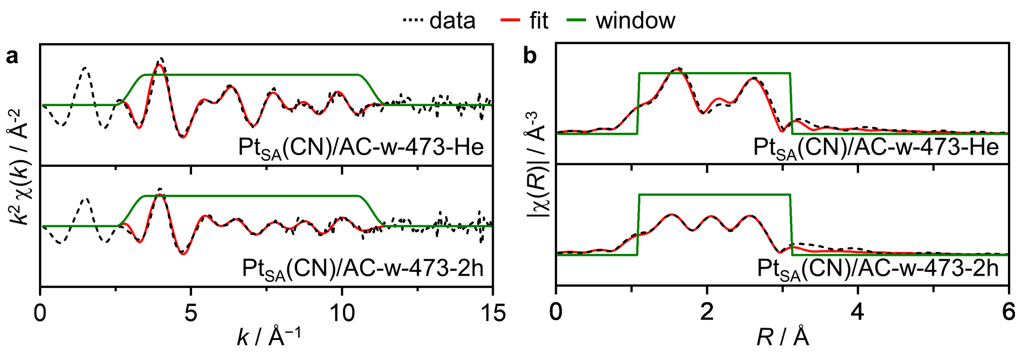

Supplementary Figure S17. a, k space and **b**, R space of experimental and fitted Pt L_3 edge EXAFS spectra of Pt$_{SA}$(CN)/AC-w-473 acquired under reaction conditions (**Supplementary Figure S26b**). Sample code: catalysts under He, **Pt(CN)$_{SA}$/AC-w-473-He**, and catalysts under reaction conditions, **Pt(CN)$_{SA}$/AC-w-473-*Y*h**. Number of hours on stream, $Y = 2$.

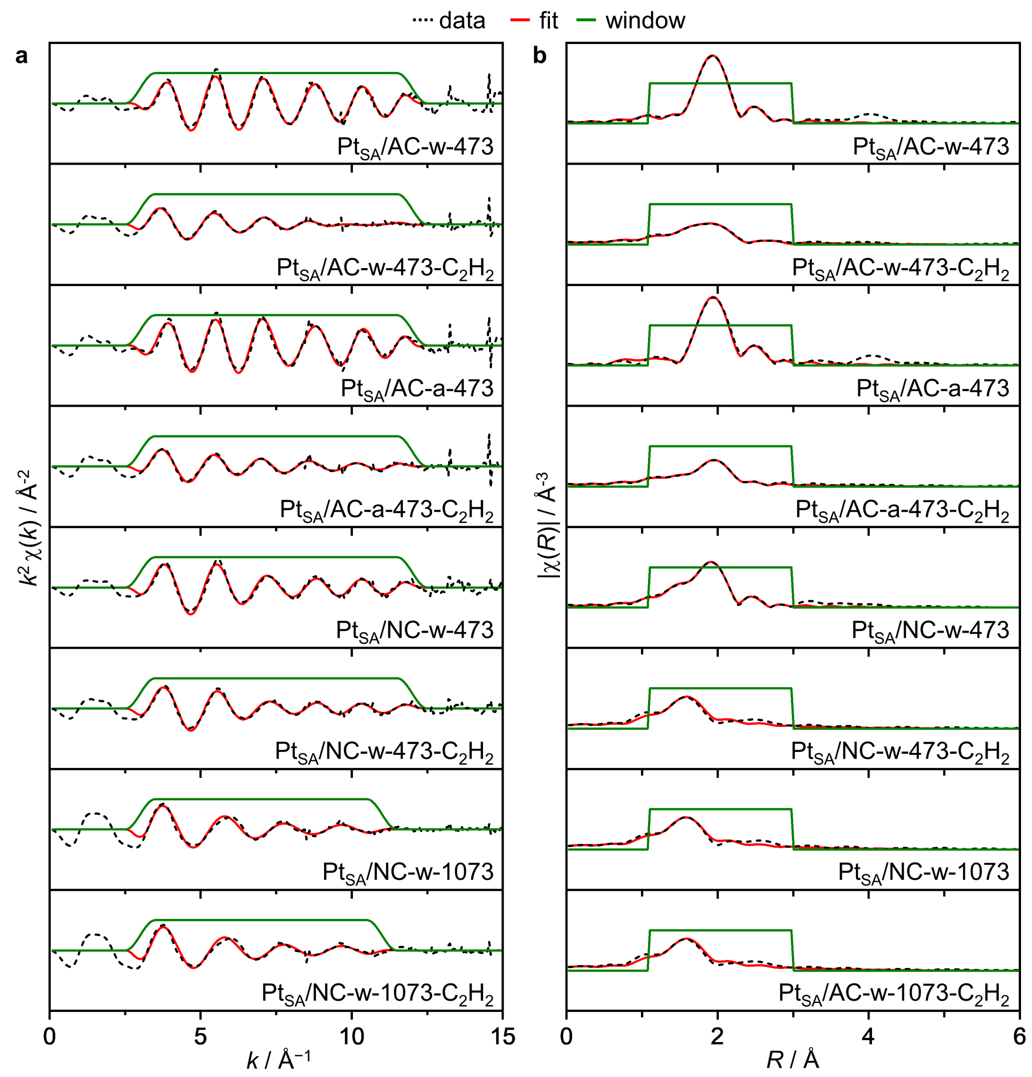

Supplementary Figure S18. a, k space and **b**, R space of experimental and fitted Pt L_3 edge EXAFS of selected Pt SACs acquired under C_2H_2 (**Supplementary Figure S26d**). Sample code: **Pt$_{SA}$/support-solvent-*T*$_a$-C$_2$H$_2$**. Support = AC or NC; solvent = w or a; activation temperature, T_a = 473-1073 K.

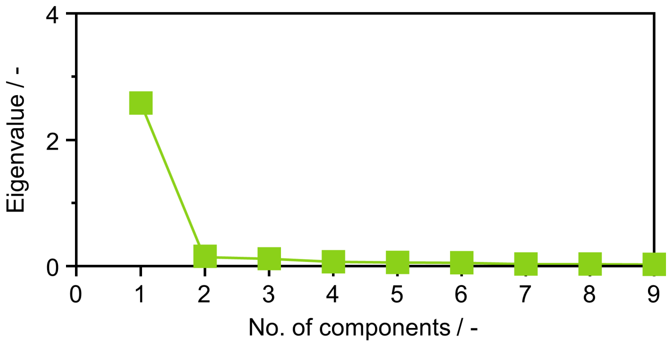

Supplementary Figure S19. Singular value decomposition analysis of Pt L_3 edge XAS spectra of Pt_{SA}/AC-w-473 acquired under C_2H_2 (**Supplementary Figure S26d**), showing that two components are needed to minimize the eigenvalue when conducting the MCR analysis. Significant reduction in the eigenvalue does not further arise from increasing the number of components.

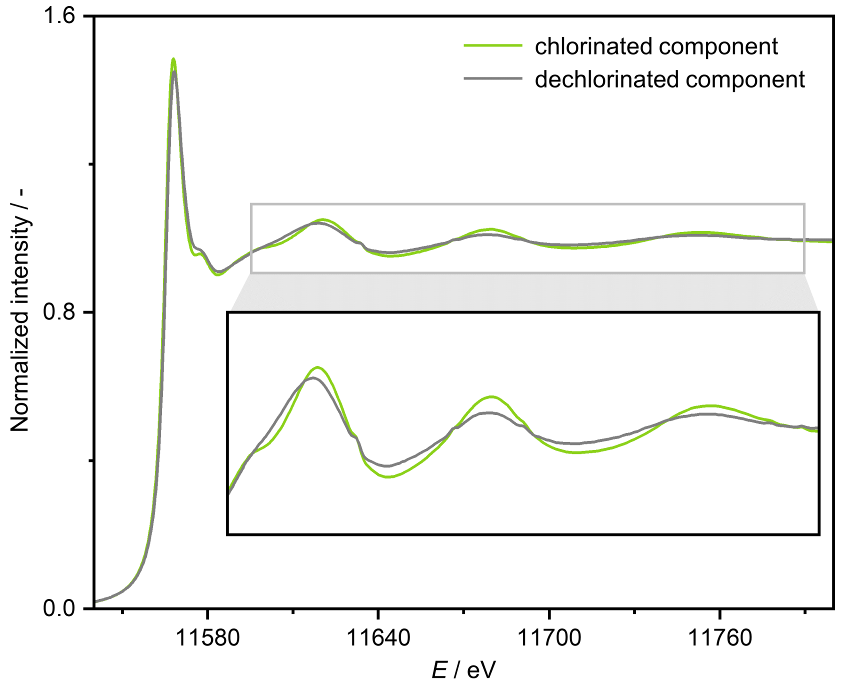

Supplementary Figure S20. Spectral components of the MCR analysis of the Pt L_3 edge XAS spectra of Pt_{SA}/AC-w-473 acquired under C_2H_2 (**Supplementary Figure S26d**).

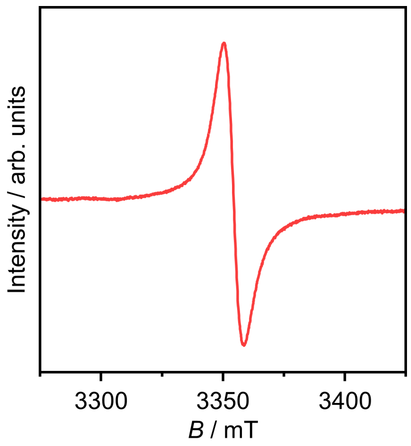

Supplementary Figure S21. CW EPR spectrum of Pt_{SA}/AC-w-473 acquired after use in acetylene hydrochlorination for 12 h.

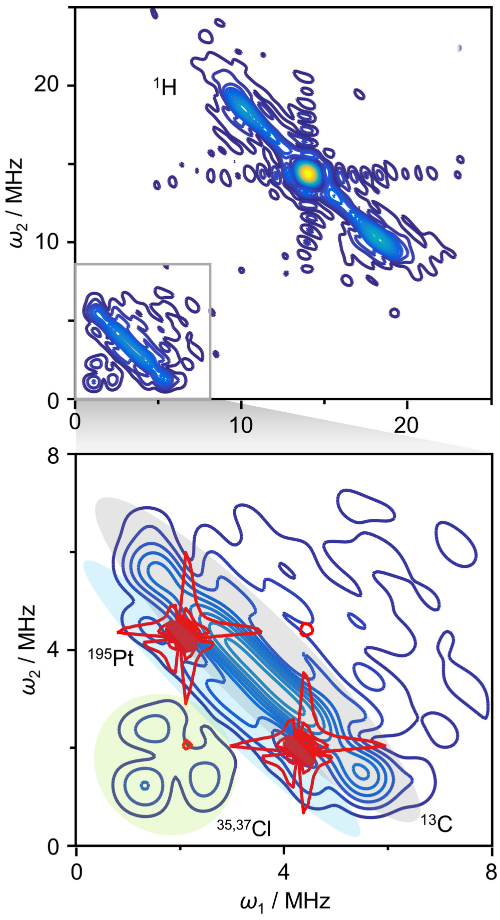

Supplementary Figure S22. Weak interaction quadrant of the 2D HYSCORE EPR spectra of carbonaceous deposits generated over Pt_{SA}/AC-w-473 in 12 h on stream. A strong coupling with ^{1}H (I = 1/2) nuclei of the polyaromatic radicals is detected (top) together with interactions with ^{13}C (I = 1/2), ^{35,37}Cl (I = 3/2), and ^{195}Pt (I = 1/2, 34% isotopic abundance) at low frequencies (bottom). Simulation of the ^{195}Pt component (red, bottom) enables determination of the spin density in the Pt *s* orbitals, as well as estimation of both the lower limit of the average coke radical-metal site distance and the upper limit for the spin density in Pt *d* orbitals. Reaction conditions: $T_{bed} = 473$ K, $F_{tot} = 15$ cm^3 min^{-1}, C_2H_2:HCl:Ar = 40:44:16, $W_{cat} = 0.25$ g.

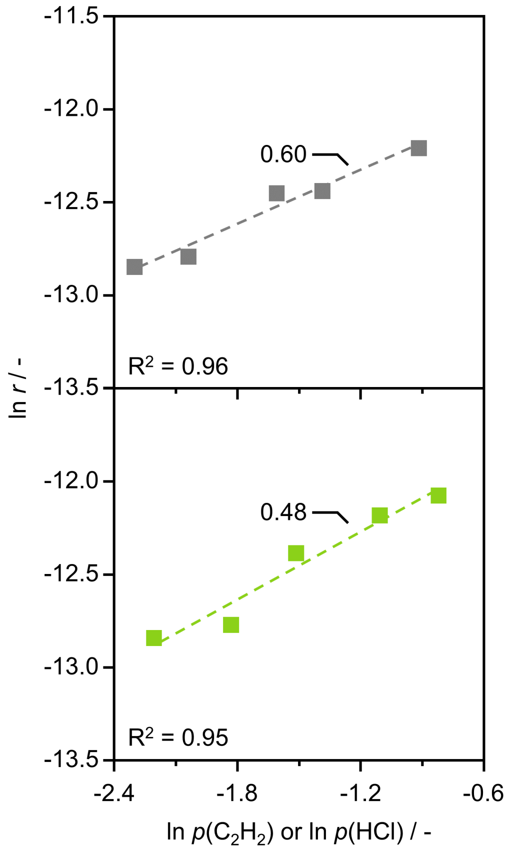

Supplementary Figure S23. Reaction rates, *r*, in acetylene hydrochlorination as a function of the inlet partial pressure of acetylene ($p(C_2H_2)$, top) or hydrogen chloride (p(HCl), bottom). The partial reaction order of both reactants, *n*, corresponds to the slope of the fitting lines, respectively. Reaction conditions: T_{bed} = 473 K, F_t = 10 cm^3 min^{-1}, $p(C_2H_2$, HCl) = 0.1-0.4 bar, W_{cat} = 0.03 g, and P = 1 bar.

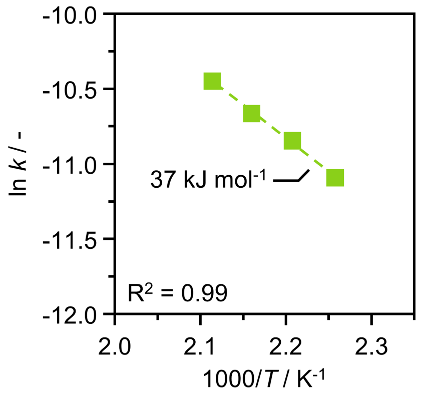

Supplementary Figure S24. Arrhenius plots of the Pt_{SA}/AC-w-473 catalyst in acetylene hydrochlorination with the corresponding apparent activation energy, E_a. Reaction conditions: T_{bed} = 473 K, F_{tot} = 10 cm^3 min^{-1}, C_2H_2:HCl:Ar = 10:11:16, W_{cat} = 0.03 g, and P = 1 bar.

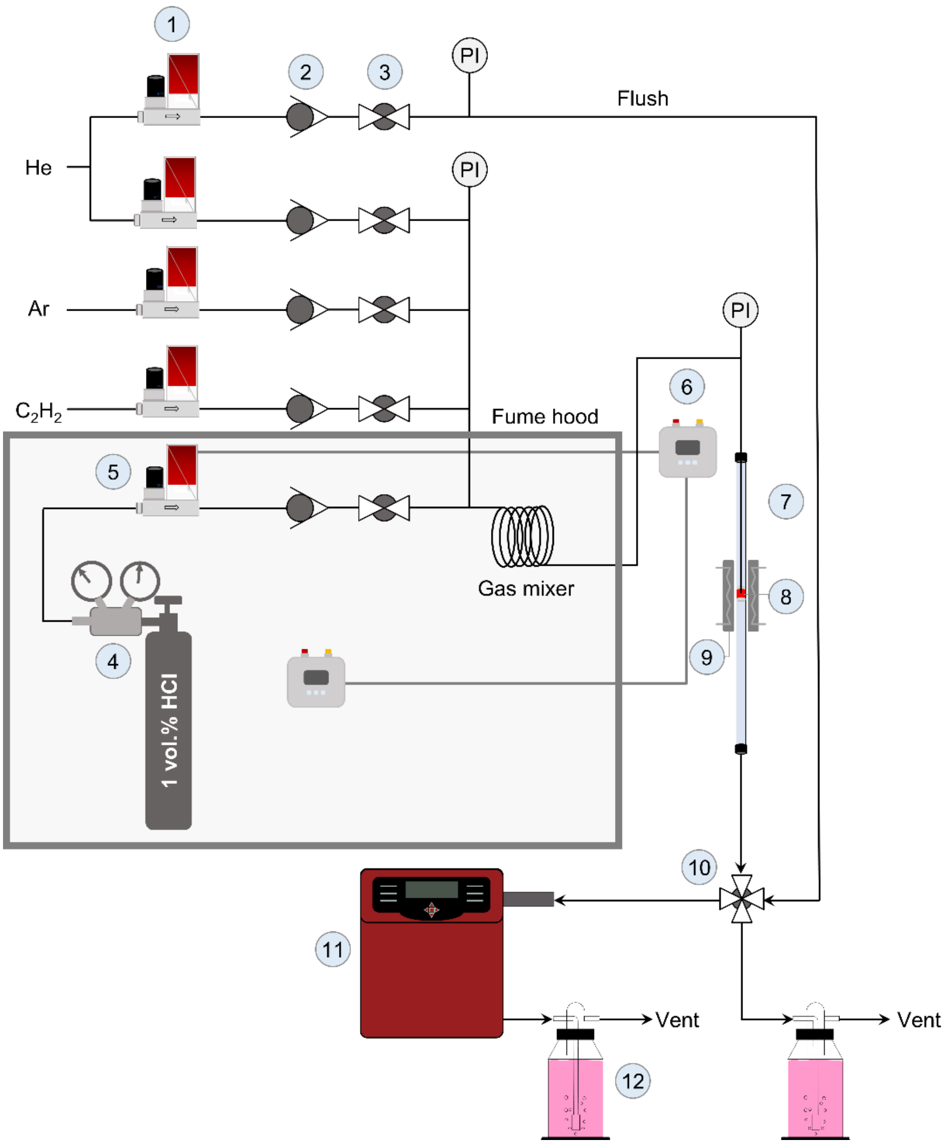

Supplementary Figure S25. Set-up used for catalyst monitoring by *operando* XAS during thermal activation and under acetylene hydrochlorination-relevant conditions. 1: He, Ar, and C_2H_2 mass-flow controllers, 2: check valves, 3: two-way on-off valves, 4: HCl gas cylinder and pressure reducer, 5: HCl mass-flow controller, 6: HCl sensor, 7: quartz capillary, 8: catalyst bed, 9: heating element, 10: four-way valve, 11: mass spectrometer, 12: NaOH scrubber, PI: pressure indicator, and TI: temperature indicator.

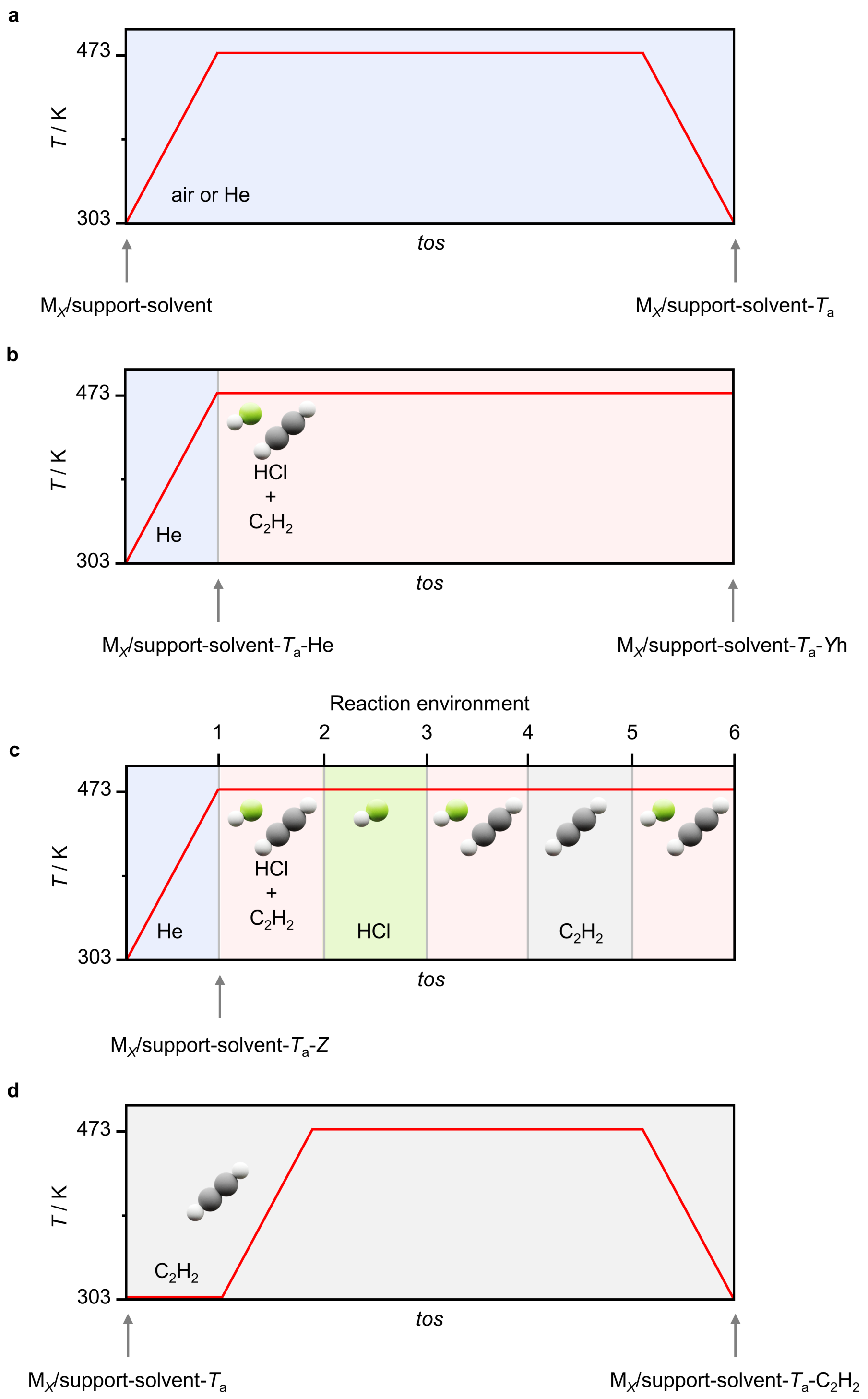

Supplementary Figure S26. Overview of catalyst monitoring conditions by *operando* XAS, as a function of temperature (*T*) and time on stream (*tos*), together with the related sample notation (**Supplementary Table S5**). **a,** Thermal activation. **b,** Acetylene hydrochlorination. **c,** Sequence of reactive environments relevant to acetylene hydrochlorination. **d,** C_2H_2 adsorption.

4. Supplementary References

1 Lin, R., Kaiser, S. K., Hauert, R. & Pérez-Ramírez, J. Descriptors for high-performance nitrogen-doped carbon catalysts in acetylene hydrochlorination. *ACS Catal.* **8**, 1114 (2018).

2 Kaiser, S. K. *et al.* Nanostructuring unlocks high performance of platinum single-atom catalysts for stable vinyl chloride production. *Nat. Catal.* **3**, 376 (2020).

3 Moulder, J. F., Stickle, W. F., Sobol, P. E. & Bomben, K. D. *Handbook of X-Ray Photoelectron Spectroscopy* 63 (Physical Electronics, Inc., 1995).

4 Zhou, J.-H. *et al.* Characterization of surface oxygen complexes on carbon nanofibers by TPD, XPS and FT-IR. *Carbon* **45**, 785 (2007).

5 Schweiger, A. & Jeschke, G. Principles of pulse electron paramagnetic resonance. *Oxford University Press, New York* (2001).

6 Morton, J. R. & Preston, K. F. Atomic parameters for paramagnetic resonance data. *J. Magn. Reson.* **30**, 3 (1969).

7 Abdala, P. M. *et al.* Scientific opportunities for heterogeneous catalysis research at the SuperXAS and SNBL beam lines. *Chimia* **66**, 699 (2012).

8 Muller, O., Nachtegaal, M., Just, J., Lutzenkirchen-Hecht, D. & Frahm, R. Quick-EXAFS setup at the SuperXAS beamline for *in situ* X-ray absorption spectroscopy with 10 ms time resolution. *J. Synchrotron Radiat.* **23**, 260 (2016).

9 Clark, A. H., Imbao, J., Frahm, R. & Nachtegaal, M. ProQEXAFS: A highly optimized parallelized rapid processing software for QEXAFS data. *J. Synchrotron Radiat.* **27**, 551 (2020).

10 Newville, M. IFEFFIT: Interactive XAFS analysis and FEFF fitting. *J. Synchrotron Radiat.* **8**, 322 (2001).

11 Camp, C. H. pyMCR: A python library for mutivalriate curve reolution analysis with alternating regression (MCR-AR). *J. Res. Natl. Inst. Radiat.* **124**, 124018 (2019).

12 Windig , W. *et al.* A new approach for interactive self-modeling mixture analysis. *Chemometr. Intell. Lab. Syst.* **77**, 85 (2005).

13 Carberry, J. J. in *Catalysis: Science and Technology* (eds J.R. Anderson & M. Boudart) Ch. 3, 131 (Springer-Verlag, 1987).

14 Mears, D. Diagnostic criteria for heat transport limitations in fixed bed reactors. *J. Catal.* **20**, 127 (1971).

15 Weisz, P. B. & Prater, C. D. Interpretation of measurements in experimental catalysis. *Adv. Catal.* **6**, 143 (1954).

16 Kresse, G. & Furthmüller, J. Efficient iterative schemes for *ab initio* total-energy calculations using a plane-wave basis set. *Phys. Rev. B* **54**, 11169 (1996).

17 Kresse, G. & Furthmüller, J. Efficiency of *ab-initio* total energy calculations for metals and semiconductors using a plane-wave basis set. *Comput. Mater. Sci.* **6**, 15 (1996).

18 Perdew, J. P., Burke, K., Ernzerhof, M. Generalized gradient approximation made simple. *Phys. Rev. Lett.* **77**, 3865 (1996).

19 Grimme, S., Antony, J., Ehrlich, S., & Krieg, H. A consistent and accurate *ab initio* parametrization of density functional dispersion correction (DFT-D) for the 94 elements H-Pu. *J. Chem. Phys.* **132**, 154104 (2010).

20 Blöchl, P. E. Projector augmented-wave method. *Phys. Rev. B.* **50**, 17953 (1994).

21 Kresse, G. & Joubert, D. From ultrasoft pseudopotentials to the projector augmented-wave method. *Phys. Rev. B.* **59**, 1758 (1999).

22 Makov, G. & Payne, M. C. Periodic boundary conditions in *ab initio* calculations. *Phys. Rev. B.* **51**, 4014 (1995).

23 Campbell, C. T., & Sellers, J. R. V. The entropies of adsorbed molecules. *J. Am. Chem. Soc.* **134**, 18109 (2012).

24 Osman, A. I. *et al.* The production and application of carbon nanomaterials from high alkali silicate herbaceous biomass. *Sci. Rep.* **10**, 2563 (2020).
